# Bifidobacterial GH146 β-l-arabinofuranosidase for the removal of β1,3-l-arabinofuranosides on plant glycans

**DOI:** 10.1007/s00253-024-13014-8

**Published:** 2024-02-07

**Authors:** Kiyotaka Fujita, Hanako Tsunomachi, Pan Lixia, Shun Maruyama, Masayuki Miyake, Aimi Dakeshita, Kanefumi Kitahara, Katsunori Tanaka, Yukishige Ito, Akihiro Ishiwata, Shinya Fushinobu

**Affiliations:** 1 Faculty of Agriculture, Kagoshima University, 1-21-24 Korimoto, Kagoshima, Kagoshima 890-0065 Japan; 2 The United Graduate School of Agricultural Sciences, Kagoshima University, 1-21-24 Korimoto, Kagoshima, Kagoshima 890-0065 Japan; 3 Department of Biotechnology, The University of Tokyo, 1-1-1 Yayoi, Bunkyo-Ku, Tokyo 113-8657 Japan; 4 National Key Laboratory of Non-food Biomass Energy Technology, Guangxi Key Laboratory of Marine Natural Products and Combinatorial Biosynthesis Chemistry, Guangxi Academy of Sciences, Nanning, 530007 China; 5 RIKEN, Cluster for Pioneering Research, 2-1 Hirosawa, Wako, Saitama 351-0198 Japan; 6 Department of Chemical Science and Engineering, Tokyo Institute of Technology, 2-12-1 Ookayama, Meguro-Ku, Tokyo 152-8552 Japan; 7 Graduate School of Science, Osaka University, 1-1 Machikaneyama-Cho, Toyonaka, Osaka 560-0043 Japan; 8 Collaborative Research Institute for Innovative Microbiology, The University of Tokyo, 1-1-1 Yayoi, Bunkyo-Ku, Tokyo 113-8657 Japan

**Keywords:** β-l-Arabinofuranosidase, *Bifidobacterium longum* subsp. *longum*, Arabinogalactan-protein, Glycoside hydrolase

## Abstract

**Abstract:**

l-Arabinofuranosides with β-linkages are present in several plant molecules, such as arabinogalactan proteins (AGPs), extensin, arabinan, and rhamnogalacturonan-II. We previously characterized a β-l-arabinofuranosidase from *Bifidobacterium longum* subsp. *longum* JCM 1217, Bll1HypBA1, which was found to belong to the glycoside hydrolase (GH) family 127. This strain encodes two GH127 genes and two GH146 genes. In the present study, we characterized a GH146 β-l-arabinofuranosidase, Bll3HypBA1 (BLLJ_1848), which was found to constitute a gene cluster with AGP-degrading enzymes. This recombinant enzyme degraded AGPs and arabinan, which contain Ara*f-*β1,3-Ara*f* structures. In addition, the recombinant enzyme hydrolyzed oligosaccharides containing Ara*f-*β1,3-Ara*f* structures but not those containing Ara*f-*β1,2-Ara*f* and Ara*f-*β1,5-Ara*f* structures. The crystal structures of Bll3HypBA1 were determined at resolutions up to 1.7 Å. The monomeric structure of Bll3HypBA1 comprised a catalytic (α/α)_6_ barrel and two β-sandwich domains. A hairpin structure with two β-strands was observed in Bll3HypBA1, to extend from a β-sandwich domain and partially cover the active site. The active site contains a Zn^2+^ ion coordinated by Cys_3_-Glu and exhibits structural conservation of the GH127 cysteine glycosidase Bll1HypBA1. This is the first study to report on a β1,3-specific β-l-arabinofuranosidase.

**Key points:**

*• β1,3-* *l* *-Arabinofuranose residues are present in arabinogalactan proteins and arabinans as a terminal sugar.*

*• β-* *l* *-Arabinofuranosidases are widely present in intestinal bacteria.*

*• Bll3HypBA1 is the first enzyme characterized as a β1,3-linkage-specific β-* *l* *-arabinofuranosidase.*

**Supplementary Information:**

The online version contains supplementary material available at 10.1007/s00253-024-13014-8.

## Introduction

l-Arabinose occurs in four distinct forms in plant polysaccharides: α- and β-l-arabinofuranose (Ara*f*) and l-arabinopyranose (Ara*p*). Although β-Ara*f* occurs in significantly less amount than α-Ara*f* in plant carbohydrate polymers, it is present as β-l-arabinooligosaccharide (β-AOS) chains in extensin (e.g., Ara*f-*β1,2-Ara*f-*β1,2-Ara*f-*β1-hydroxyproline (Hyp); Ara_3_-Hyp). β-Ara*f* is also present as a terminal sugar in plant polysaccharides, e.g., terminal β1,2- and β1,5-Ara*f* are present in the complicated sugar chain of rhamnogalacturonan-II (RG-II) in pectin (Pellerin et al. 1996). Moreover, terminal Ara*f-*β1,3-Ara*f*-α1 structures are found in several plant polysaccharides, including arabinan from sugar beet (Wefers et al. 2017) and quinoa seeds (Wefers et al. 2014), arabinoxylan from green leaves of Lauraceae (Das et al. 2013), arabinoxyloglucan from tomato cultured cells (York et al. 1996), and arabinogalactan proteins (AGPs) from rice anthers (Kawaguchi et al. 1996). β-Ara*p* is also present as a terminal sugar in plant polysaccharides, including type-II arabinogalactan (AG) from larch (Ponder and Richards 1997), AGPs from wheat (Tryfona et al. 2010), and gum arabic (Tischer et al. 2002). α-Ara*p* is found only as a constituent sugar of RG-II in plant polysaccharides (Pellerin et al. 1996).

Pfam DUF1680 (PF07944), which was renamed to Glyco_hydro_127, contains 24,000 proteins that are distributed among 7482 bacterial, fungal, and plant species. HypBA1 (Bll1HypBA1; encoded by BLLJ_0211) is the first characterized GH127 β-l-arabinofuranosidase (EC 3.2.1.185) (Fujita et al. 2014b). Bll1HypBA1 from *Bifidobacterium longum* subsp. *longum* JCM 1217 degrades Ara*f-*β1,2-Ara released from Ara_3_-Hyp by GH121 β-l-arabinobiosidase (HypBA2: BLLJ_0212) (Fujita et al. 2011). Crystallographic studies of Bll1HypBA1 have revealed that a cysteine residue serves as the catalytic nucleophile (Ishiwata et al. 2022b; Ito et al. 2014; Maruyama et al. 2022; McGregor et al. 2021). In addition to GH137 and GH142 β-l-arabinofuranosidases for RG-II degradation in *Bacteroides thetaiotaomicron* (Ndeh et al. 2017), GH146 was established after characterization of BT0349 as an β-l-arabinofuranosidase for arabinan-derived arabinotetraose and β1,2-l-arabinobiose (Luis et al. 2018). We also characterized a GH146 member, XCV2724, from *Xanthomonas euvesicatoria* (XeHypBA1) (Nakamura et al. 2018). This enzyme hydrolyzed Ara*f-*β1-Hyp predominately and partially for Ara*f-*β1,2-Ara (Ishiwata et al. 2023). In addition to two GH127 members (Bll1HypBA1 and BLLJ_1826), *B. longum* subsp. *longum* JCM 1217 encodes two GH146 members (BLLJ_1848 and BLLJ_0089). BLLJ_0089 was recently characterized as a β-l-arabinofuranosidase (Bll4HypBA1) for Ara*f-*β1-linked Hyp on the backbone of Hyp-rich glycoprotein (HRGP) (Ishiwata et al. 2023). At present, the three-dimensional structure of only one enzyme (BT0349) in the GH146 family has been reported (Luis et al. 2018; McGregor et al. 2021). In the present study, we characterized the recombinant BLLJ_1848 as a β-1,3-specific GH146 β-l-arabinofuranosidase (Bll3HypBA1) using natural and synthetic substrates. In addition, we performed X-ray crystallography of Bll3HypBA1.

## Materials and methods

### Substrates

**Fig. 1:**
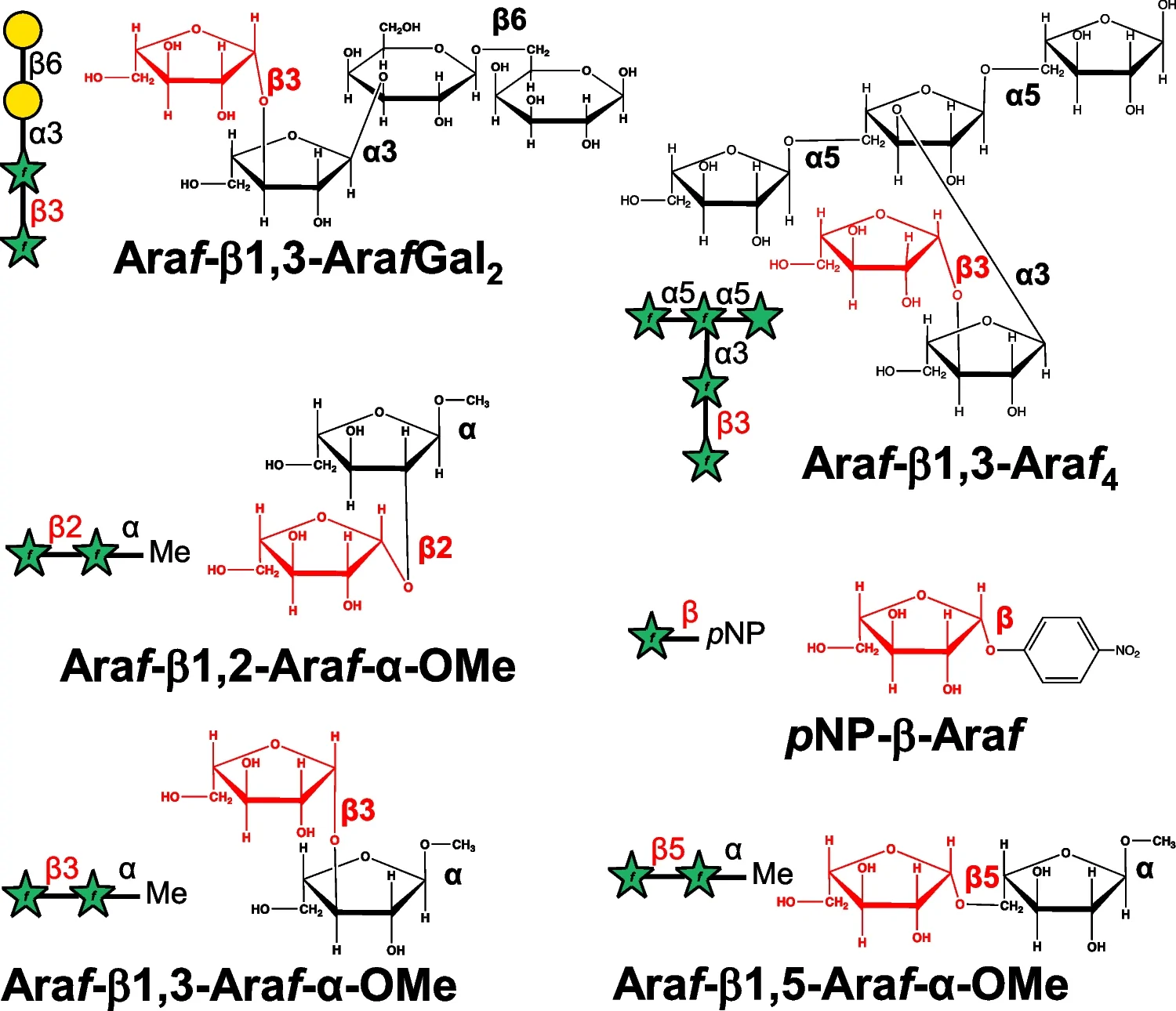
Chemical and schematic structures of the substrates used in this study. β-l-Ara*f*-structure is highlighted in red. Symbols: d-galactose, yellow circle; l-arabinose, green star

Larch wood AG was purchased from Tokyo Chemical Industry Co., Ltd. (Tokyo, Japan). Gum arabic from *Acacia senegal* was purchased from Sigma-Aldrich (St. Louis, MO, USA). *p*-Nitrophenyl (*p*NP) β-l-arabinofuranoside was synthesized as described in a previous study (Kaeothip et al. 2013a). Young rice panicles, cultivar Nikomaru, were harvested from experimental farms at the Faculty of Agriculture, Kagoshima University (Kagoshima, Japan). According to a previous study (Kawaguchi et al. 1996), l-Ara*f-*β1,3-l-Ara*f-*α1,3-Gal*-*β1,6-Gal (Ara*f*-β1,3-Ara*f*Gal_2_) and AGP from rice anthers were prepared from young rice panicles. l-Ara*f-*α1,5-[l-Ara*f-*β1,3-l-Ara*f-*α1,3-]-l-Ara*f-*α1,5-l-Ara (Ara*f*-β1,3-Ara*f*_4_) from quinoa seed arabinan was prepared through endo-1,5-α-l-arabinanase treatment as described by Wefers et al. (Wefers et al. 2014). These oligosaccharides were further purified using HPLC on a Cosmosil PBr column (Nacalai Tesque Inc., Kyoto, Japan) as described previously (Sasaki et al. 2021). The fractions containing oligosaccharides were collected and analyzed using MALDI-TOF MS (Bruker Daltonics, Leipzig, Germany).

l-Ara*f-*β1,2-l-Ara*f-*α1-OMe (Ara*f*-β1,2-Ara*f*-α-OMe), l-Ara*f-*β1,3-l-Ara*f-*α1-OMe (Ara*f*-β1,3-Ara*f*-α-OMe), and l-Ara*f-*β1,5-l-Ara*f-*α1-OMe (Ara*f*-β1,5-Ara*f*-α-OMe) were previously prepared stereospecifically (Ishiwata et al. 2022a) via 1,2-*cis* selective β-arabinofuranosylation using 2-naphthylmethyl ether-mediated intramolecular aglycon delivery (Ishiwata and Ito 2011; Ishiwata et al. 2014, 2008; Kaeothip et al. 2013b). β-l-Arabinooligosaccharides were prepared according to previous reports (Fujita et al. 2014b). Other chemicals were purchased from Fujifilm Wako Pure Chemical Industries Ltd. (Osaka, Japan). Ara*f*-β1,3-Ara*f*Gal_2_ was labeled with *p*-aminobenzoic ethyl ester (ABEE) as described by Wang (Wang et al. 1984). The ABEE derivative was purified on a Cosmosil PBr column with a linear gradient of CH_3_CN/water from 0:100 to 70:30 (v/v) for 30 min at a constant flow rate of 4.7 mL/min at 30 °C. Elution was monitored using a fluorescence detector (FP-2020, JASCO, Japan) with Ex/Em of 305/360 nm. Figure 1 depicts the chemical structures of the substrates used in this study.

### Enzymes

β-l-Arabinofuranosidases Bll1HypBA1 (Fujita et al. 2014b), Bll2HypBA1, and Bll4HypBA1 (Ishiwata et al. 2023) enzymes were prepared according to previous reports. GH43_24 exo-β-1,3-galactanase Bl1,3Gal (Fujita et al. 2014a) and GH43_22 α-l-arabinofuranosidase BlArafA (Fujita et al. 2019a) were prepared as described previously. Endo-1,5-α-l-arabinanase (AnEARAB) and α-l-arabinofuranosidase (AnAFASE) from *Aspergillus niger* were purchased from Megazyme (Bray, Ireland). Table 1 shows the enzyme list used in this study.

**Table 1:** Enzymes used in this study

Enzyme name (locus tag)	Family	Activity
Bll1HypBA1 (BLLJ_0211)	GH127	β-l-Arabinofuranosidase
Bll2HypBA1 (BLLJ_1826)	GH127	β-l-Arabinofuranosidase
Bll3HypBA1 (BLLJ_1848)	GH146	β-l-Arabinofuranosidase
Bll4HypBA1 (BLLJ_0089)	GH146	β-l-Arabinofuranosidase
BlArafA (BLLJ_1854)	GH43_22	α-l-Arabinofuranosidase
Bl1,3Gal (BLLJ_1840)	GH43_24	Exo-β-1,3-galactanase
AnAFASE	GH51	α-l-Arabinofuranosidase
AnEARAB	GH43	Endo-1,5-α-l-arabinanase

### Expression and purification of recombinant Bll3HypBA1

**Fig. 2:**
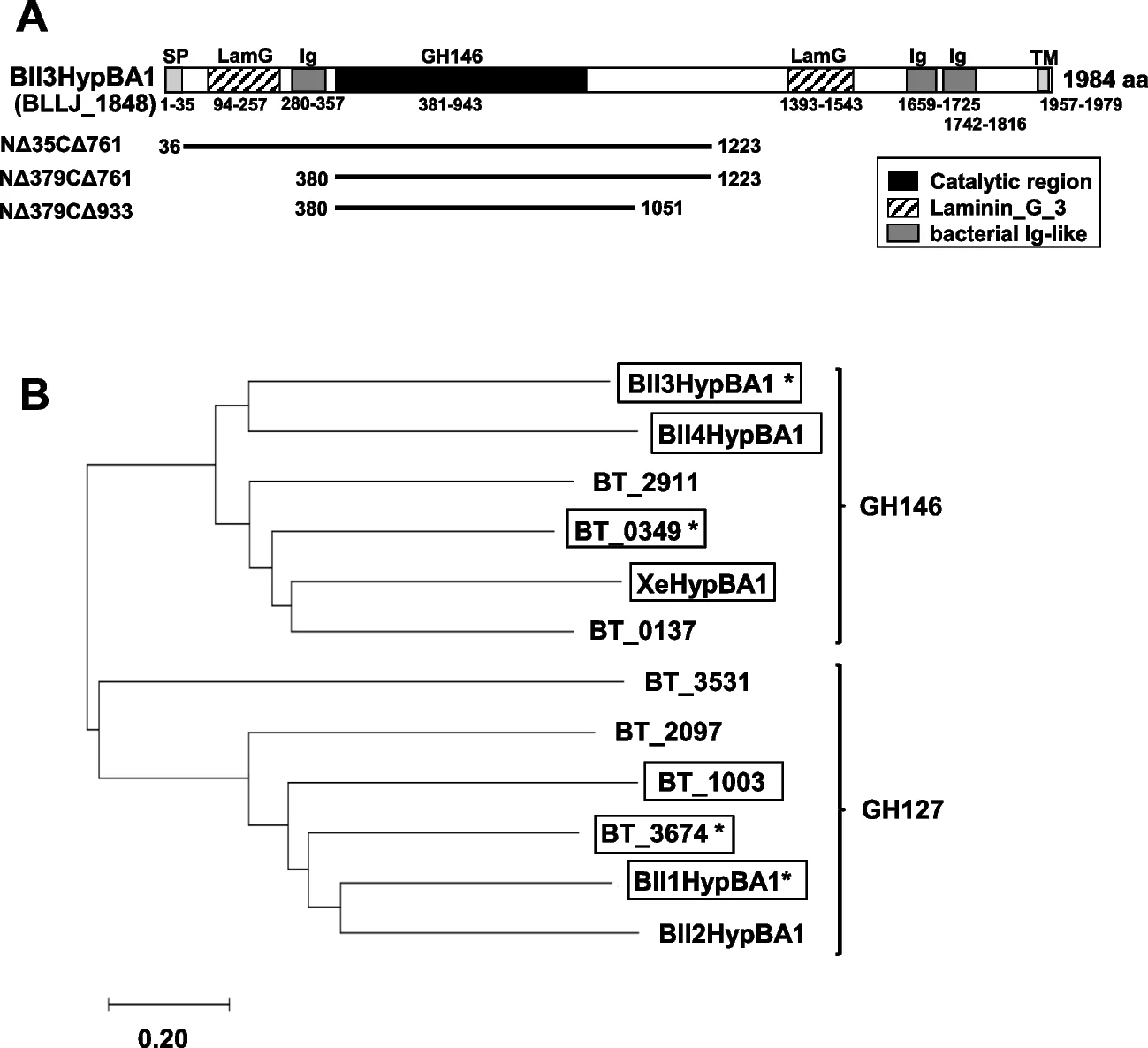
Sequence features of Bll3HypBA1 and GH127/146 β-l-arabinofuranosidases. **A** Domain structure of Bll3HypBA1. Domain structures were predicted using SignalP5.0 (https://services.healthtech.dtu.dk/services/SignalP-5.0/) and InterPro (https://www.ebi.ac.uk/interpro/) servers. The domains were indicated as follows: signal peptide (SP), laminin_G_3 (LamG), bacterial Ig-like domain (Ig), and transmembrane region (TM). Here, the lines indicate the expression regions of the Bll3HypBA1 recombinant proteins. **B** The phylogenetic tree of GH146 and GH127 β-l-arabinofuranosidase members in *Bacteroides thetaiotaomicron* VPI-5482, *X. euvesicatoria*, and *B. longum* subsp. *longum* JCM 1217. The phylogenetic tree was constructed with the MEGAX software. Enzymes characterized previously or in this study are enclosed in the box. Here, the asterisk (*) indicates enzymes for which crystallographic studies have been performed. Almost all enzymes were characterized as β-l-arabinofuranosidase, except for BT1003 (aceric acid hydrolase). GenBank accession numbers are shown alongside the characterized enzymatic names and/or protein locus tags as follows: Bll1HypBA1/BLLJ_0211 (BAJ65881), Bll2HypBA1/BLLJ_1826 (BAJ67491), Bll4HypBA1/BLLJ_0089 (BAJ65759), XeHypBA1/XCV2724 (CAJ24403), BT_0349 (AAO75456), BT_2911 (AAO78017), BT_0137 (AAO75244), BT3531 (AAO78637), BT2097 (AAO77204), BT1003 (AAO76110), BT3674 (AAO78779)

The genomic DNA of *B. longum* subsp. *longum* JCM 1217 was subjected to PCR amplification of the Bll3HypBA1 gene. The forward (5′-AGGAGATATACCATGGCAGAAGTGGACTCCAGCA-3′) and reverse (5′-TGCTCGAGTGCGGCCGCGTTCTGCATGCGCAC-3′) primers were designed using nucleotides 106–124 and 3655–3671 from the Bll3HypBA1 gene. The underlined texts indicate complementary nucleotides to the template. The PCR amplification of Bll3HypBA1-NΔ35CΔ761, which encodes amino acids (aa) 36–1223, was designed to eliminate the N-terminal signal peptide and C-terminal domains (Fig. 2A). The amplicon was cloned into the pET-23d vector (Novagen, Madison, WI, USA) using the In-Fusion HD Cloning Kit (Clontech Laboratories Inc., Palo Alto, CA, USA). The KOD-plus mutagenesis kit (Toyobo Co., Ltd., Osaka, Japan) and the primers listed in Table S1 were used to generate the Bll3HypBA1 deletion mutants. The primers NΔ reverse and NΔ379 forward were used to construct Bll3HypBA1-NΔ379CΔ761 (aa: 380–1223), whereas the primers CΔ forward and CΔ933 reverse were used to construct Bll3HypBA1-NΔ379CΔ933 (aa: 380–1051) (Fig. 2A). The constructed plasmids were sequenced and transformed into *E. coli* BL21 (λDE3) cells, which were then grown at 20 °C using the Overnight Express Autoinduction System (Novagen). The His-tagged proteins were extracted using BugBuster protein extraction reagent (Novagen), purified on a TALON metal affinity resin (Clontech Laboratories Inc.), and then desalted and concentrated using an ultrafiltration membrane (10-kDa cut-off; Millipore Co., Billerica, MA, USA). The sequence of the Bll3HypBA1-NΔ35CΔ761 gene was deposited in the DDBJ database under the accession number LC765464.

### Thin-layer chromatography (TLC), high-performance anion-exchange chromatography with pulsed amperometric detection (HPAEC-PAD), and HPLC

For TLC, silica gel 60 aluminum plates (Merck KGaA, Darmstadt, Germany) were used with a 7:1:2 (v/v/v) of 1-propanol/EtOH/water solvent mixture and then visualized by spraying orcinol-sulfate on the plates (Holmes and O'Brien 1979). For HPAEC-PAD, oligosaccharides were analyzed using a CarboPac PA-1 column (Dionex Corp., Sunnyvale, CA, USA) as described previously (Sasaki et al. 2021). For HPLC, ABEE-labeled Ara*f*-β1,3-Ara*f*Gal_2_ and reaction products were analyzed by a Cosmosil Sugar-D (Nacalai Tesque Inc.) as described previously (Ishiwata et al. 2022b).

### pH and temperature dependence of Bll3HypBA1 activity

The hydrolytic activity of Bll3HypBA1-NΔ35CΔ761 was assayed using Ara*f*-β1,3-Ara*f*Gal_2_-ABEE as the substrate and 50 mM sodium acetate (pH 3.5–6.0) and 50 mM sodium phosphate (pH 6.5–8.0) as buffers. The substrate (25 µM) was incubated with 0.05 µg/ml of Bll3HypBA1-NΔ35CΔ761 in 40 µL of each buffer (50 mM) at 40 °C for 20 min. The reaction was stopped by adding 10 µL of 5% trichloroacetic acid (TCA) and then analyzed via HPLC. Next, the effect of temperature on enzyme activity was examined using 50 mM sodium acetate buffer (pH 5.5) at 25–60 °C.

### Substrate specificities of Bll3HypBA1 toward polysaccharides

The hydrolytic activity of Bll3HypBA1-NΔ35CΔ761 was analyzed using sugar beet arabinan, larch AG, and gum arabic AGP as polysaccharide substrates. These substrates (1.0%) were incubated with 0.1 µg/mL of Bll3HypBA1-NΔ35CΔ761, Bll1HypBA1, Bll2HypBA1, or Bll4HypBA1 in 40 µL of 50 mM sodium acetate buffer (pH 5.5) at 40 °C for 16 h. Following incubation, the released l-arabinose was analyzed using TLC, as described previously. For the comparison of the specific activity of Bll3HypBA1 with or without N-terminal LamG domain, the substrates (1.0%) were incubated with 0.1 µg/ml of Bll3HypBA1-NΔ35CΔ761 and Bll3HypBA1-NΔ379CΔ761 in 40 µL of 50 mM sodium acetate buffer (pH 5.5) at 40 °C for 2 h. The reaction was terminated by boiling, and the liberated l-arabinose was analyzed using HPAEC-PAD.

### Combination reactions of Bll3HypBA1 and AGP degradative enzymes

Bll3HypBA1-NΔ35CΔ761 (0.1 µg/ml) reacted with Ara*f*-β1,3-Ara*f*Gal_2_ and Ara*f*-β1,3-Ara*f*_4_ with or without BlArafA/AnAFASE at 40 °C for 16 h. The reaction mixtures were analyzed via HPAEC-PAD and MALDI-TOF MS. To determine the mode of action of rice AGP, the following reactions involving Bll3HypBA1-NΔ35CΔ761, BlArafA, and Bl1,3Gal were conducted: 1.0% rice AGP were incubated with enzymes (0.1 µg/mL each) in a 50 mM sodium phosphate buffer (pH 6.0) at 37 °C for 16 h. The reaction products were analyzed via HPAEC-PAD.

### Substrate specificities of Bll3HypBA1 toward oligosaccharides

Next, the substrate specificities of Bll3HypBA1 were analyzed using synthetic and natural substrates. Since the region following residue 1052 in the NΔ379CΔ761 was not affected to structure of the catalytic region, Bll3HypBA1-NΔ379CΔ761 or CΔ933 were used for the analysis of substrate specificities. Synthetic Ara*f*-β1,2-Ara*f*-α-OMe, Ara*f*-β1,3-Ara*f*-α-OMe, and Ara*f*-β1,5-Ara*f*-α-OMe were incubated with 0.1 µg/ml of the Bll3HypBA1-NΔ379CΔ933 or Bll1HypBA1 in 40 µL of 50 mM sodium acetate buffer (pH 5.5) at 37 °C for 16 h. The reaction products were analyzed using TLC as previously described. To determine specific activities, natural oligosaccharides containing terminal Ara*f*-β1,3 and Ara*f*-β1,2 and synthetic Ara*f*-β-*p*NP and Ara*f-*β-OMe were used. Ara*f*-β1,3-Ara*f*Gal_2_ (0.15 mM) and Ara*f*-β1,3-Ara*f*_4_ (0.15 mM) were incubated with 0.01 µg/mL of Bll3HypBA1-NΔ379CΔ761 in 40 µL of 50 mM sodium acetate buffer (pH 5.5) at 40 °C for 20 min. Ara*f*-β-*p*NP (1.0 mM) was incubated with 0.1 µg/mL of Bll3HypBA1-NΔ379CΔ761 in 40 µL of 50 mM sodium acetate buffer (pH 5.5) at 40 °C for 2 h. A final concentration of 0.35 mM Ara*f*-β1,2-Ara, 0.40 mM Ara*f*-β-OMe, 0.11 mM Ara*f*-β-Hyp (*cis*), 0.11 mM Ara*f*-β-Hyp (*trans*), 0.13 mM Ara*f*-β1,2-Ara*f*-β-Hyp, and 1.0 mM Ara*f*-β1,2-Ara*f*-β1,2-Ara*f*-β-Hyp were incubated with 12 µg/ml of the Bll3HypBA1-NΔ379CΔ761 in 40 µL of 50 mM sodium acetate buffer (pH 5.5) at 40 °C for 16 h. The reactions were terminated by adding 10 µL of 5% TCA and subsequently analyzed using HPAEC-PAD. One unit of enzyme activity was defined as the amount of enzyme required to produce 1 µmol l-arabinose per minute.

### Transglycosylation activities

Transglycosylation reactions were performed using Ara*f*β1,3-AraGal_2_ as the donor and the 1-alkanols as acceptors. The substrate was incubated with 0.30 µg/mL of Bll3HypBA1-NΔ35CΔ761 in 40 µL of 50 mM sodium acetate buffer (pH 5.5) containing 5% methanol as the acceptor. After 1 h of incubation at 37 °C, the reaction was terminated by boiling. For ethanol and 1-propanol, the reactions were terminated after 3 h. TLC was used for confirmation of the reaction products as described above.

### Protein purification for crystallization

For crystallization, selenomethionine (SeMet)-labeled and native proteins were expressed in *E. coli* BL21-CodonPlus (DE3)-RP-X and BL21-CodonPlus (DE3)-RIL (Agilent Technologies, Santa Clara, CA, USA), respectively. For the expression of the SeMet-labeled protein, the transformants were grown in Se-Met core medium supplemented with 10 g/L d-glucose, 250 mg/L MgSO_4_•7H_2_O, 4.2 mg/L FeSO_4_•7H_2_O, 10 ml/L Kao and Michayluk Vitamin Solution (Sigma-Aldrich), and 25 mg/L seleno-l-methionine. For the expression of the native protein, lysogeny broth was used. Transformants were grown at 37 °C for 2 h in a medium supplemented with 50 µg/mL chloramphenicol and 100 µg/mL ampicillin. Protein expression was induced by adding 0.1 mM isopropyl-β-d-thiogalactopyranoside and 0.5 mM ZnSO_4_ to the medium, and cells were further cultured at 18 °C for 24 h. Cells were harvested via centrifugation, suspended in 20 mM Tris–HCl (pH 7.5) and 300 mM NaCl (buffer A), and later disrupted via sonication; the supernatant was then purified via serial column chromatography. Ni-immobilized metal affinity chromatography was conducted using cOmplete™ His-Tag Purification Resin (Sigma-Aldrich) with wash and elution steps of 20 mM and 300 mM imidazole in buffer A, respectively. The buffer of the eluted protein sample was exchanged with 20 mM Tris–HCl (pH 7.5) (buffer B) using Vivaspin Turbo 15 (50-kDa cut-off; Sartorius Stedim Biotech, Göttingen, Germany). The protein sample was applied to a HiTrap Q HP column (5 mL) (Cytiva, Marlborough, MA, USA) equilibrated with buffer B and eluted with a linear gradient of 0 to 500 mM NaCl. Then, the sample was concentrated using Vivaspin Turbo 15 MWCO 50,000, and the solution was changed to 20 mM Tris–HCl (pH 7.5) and 150 mM NaCl (buffer C). Subsequently, gel filtration chromatography was conducted using a HiLoad 16/60 Superdex 200 pg column (Cytiva) equilibrated with buffer C at a flow rate of 1 mL/min. The purified protein was concentrated again using Vivaspin Turbo 15, and the solution was changed to buffer B. Protein concentrations were determined using a BCA protein assay kit (Thermo Fisher Scientific, Waltham, MA, USA) with bovine serum albumin as the standard.

### Crystallography

The protein crystals were grown at 20 °C using the hanging drop vapor-diffusion method by combining the protein solution with an equal volume of a reservoir solution. SeMet-labeled Bll3HypBA1-NΔ379CΔ761 was crystallized using a 17 mg/mL protein solution and a reservoir solution containing 12% PEG 8000, 0.2 M MgCl_2_, and 0.1 M Tris–HCl (pH 8.5). The native Bll3HypBA1-NΔ379CΔ761 protein was crystallized using a protein solution (10 mg/mL) and a reservoir solution containing 15% PEG 8000, 0.2 M MgCl_2_, and 0.1 M Tris–HCl (pH 8.5). The crystals were cryoprotected in a reservoir solution supplemented with 25% glycerol. The native Bll3HypBA1-NΔ379CΔ933 protein was crystallized using a protein solution (14 mg/mL) and a reservoir solution containing 45% MPD, 0.2 M ammonium acetate, and 0.1 M HEPES–NaOH (pH 7.5). The crystal was soaked in a reservoir solution supplemented with 100 mM l-arabinose before cryocooling, but no electron density for l-arabinose was detected in the crystal structure. The crystals were flash-cooled by dipping into liquid nitrogen. X-ray diffraction data were collected at 100 K on beamlines at the Photon Factory of the High Energy Accelerator Research Organization (KEK, Tsukuba, Japan). Preliminary diffraction data were collected at SPring-8 (Hyogo, Japan). The datasets were processed using XDS (Kabsch 2010) and Aimless (Evans and Murshudov 2013). Phase determination and automated model building were performed for the data of SeMet-labeled crystal using the AutoSol pipeline of PHENIX (Adams et al. 2010). Manual model rebuilding and crystallographic refinement were performed using Coot (Emsley et al. 2010) and Refmac5 (Murshudov et al. 2011). Molecular graphic images were prepared using PyMOL (Schrödinger, LLC, New York, NY, USA).

### Site-directed mutagenesis and activity assay

Site-directed mutants of Bll3HypBA1 were constructed using the PrimeSTAR mutagenesis basal kit (TaKaRa Bio, Ohtsu, Japan) with the primers shown in Table S1 and the pET23d-Bll3HypBA1-NΔ379CΔ933 plasmid as the template. These mutant enzymes were expressed and purified using the same procedures for Bll3HypBA1-NΔ379CΔ933. We also constructed a C805A mutant; however, it did not express in *E. coli*. The activity of the wild-type enzyme (NΔ379CΔ933) and mutants was assayed via TLC using Ara*f*-β1,3-Ara*f*-α-OMe as the substrate. The assay solution consisted of 12.5 mM Ara*f*-β1,3-Ara*f*-α-OMe, 0.0125 mg/mL enzyme, 10 mM dithiothreitol, and 20 mM sodium acetate buffer (pH 4.5). After incubation at 37 °C for 30 min, the reaction was stopped by heating the sample at 95 °C for 10 min. Then, 2 µL of the sample was spotted on a TLC plate (TLC Silica gel 60 F_254_, Merck) and developed using a 2:1:1 solvent mixture (v/v/v) of ethyl acetate/acetic acid/water. The separated sugars were visualized by spraying and heating with phosphomolybdic acid in ethanol on the plates. For mutants at Zn-coordinating and catalytic residues (E694Q, E694A, E723Q, E723A, C725S, C725A, C804S, C805S, and C805A) and E694Q/C804S double mutant, we determined specific activities via HPLC using Ara*f*-β1,3-Ara*f*Gal_2_-ABEE as described above.

## Results

### Sequence features of Bll3HypBA1

Bll3HypBA1 (BLLJ_1848) contains a GH146 catalytic domain, a signal peptide, two laminin_G_3 (LamG) domains, which belong to the concanavalin A-like lectin/glucanase superfamily, three bacterial Ig-like domains, and a transmembrane domain (Fig. 2A). Furthermore, these features are shared with other cell surface anchoring glycosidases of *B. longum* subsp. *longum* JCM 1217, such as β-l-arabinobiosidase HypBA2 (BLLJ_0212) (Fujita et al. 2011), GH43 α-l-arabinofuranosidases HypAA (BLLJ_0213), BlArafA (BLLJ_1854) (Fujita et al. 2019a), BlArafB (BLLJ_1853), BlArafC (BLLJ_1852) (Komeno et al. 2022), BlArafD (BLLJ_1851) (Komeno et al. 2022), BlArafE (BLLJ_1850) (Sasaki et al. 2022), GH43_24 exo-β-1,3-galactanase Bl1,3Gal (BLLJ_1840) (Fujita et al. 2014a), and GH30_5 exo-β-1,6-galactobiohydrolase Bl1,6Gal (BLLJ_1841) (Fujita et al. 2019a). The aa sequence of the catalytic domain of Bll3HypBA1 exhibits 30% identity with the other GH146 paralog Bll4HypBA1, a β-l-arabinofuranosidase for Ara*f-*β1-linked Hyp on the HRGP backbone (Ishiwata et al. 2023). In addition, Bll3HypBA1 exhibits 31% and 28% identities with the other characterized GH146 β-l-arabinofuranosidases BT0349 from *Bacteroides thetaiotaomicron* (Luis et al. 2018) and XeHypBA1 from *X. euvesicatoria* (Nakamura et al. 2018), respectively. Furthermore, the enzymes GH146 and GH127 are separated in the phylogenetic tree (Fig. 2B), and Bll3HypBA1 shows only 23% and 19% identities with the GH127 β-l-arabinofuranosidase paralogs Bll1HypBA1 and Bll2HypBA1 (BLLJ_1826), respectively. The alignment of the GH127 and GH146 catalytic domains revealed that the Zn-coordinating residues and catalytic residues are conserved in Bll3HypBA1 (Fig. S1).

### Production of recombinant Bll3HypBA1 protein

The recombinant Bll3HypBA1-NΔ35CΔ761 protein without the N-terminal signal peptide and the C-terminal domains (Fig. 2A) was detected as a soluble protein only at a low expression level under induction condition at 20 °C. The purified recombinant Bll3HypBA1-NΔ35CΔ761 protein migrated as a single band on SDS-PAGE, which was in agreement with the theoretical molecular mass of 129,964 Da (Fig. S2). To obtain a clone suitable for X-ray crystallography and biochemical analysis, we constructed Bll3HypBA1-NΔ379CΔ761 and Bll3HypBA1-NΔ379CΔ933 without the LamG region. These clones were expressed as soluble proteins at 30 °C and purified as a single band in agreement with the theoretical molecular mass of 94,145 Da for Bll3HypBA1-NΔ379CΔ761 and 75,016 Da for Bll3HypBA1-NΔ379CΔ933 (Fig. S2). The estimated molecular masses by gel filtration chromatography were 91 kDa and 76 kDa for Bll3HypBA1-NΔ379CΔ761 and Bll3HypBA1-NΔ379CΔ933, respectively, suggesting that both protein constructs are monomeric in solution (data not shown).

### Substrate specificity and general properties of the recombinant Bll3HypBA1 protein

**Fig. 3:**
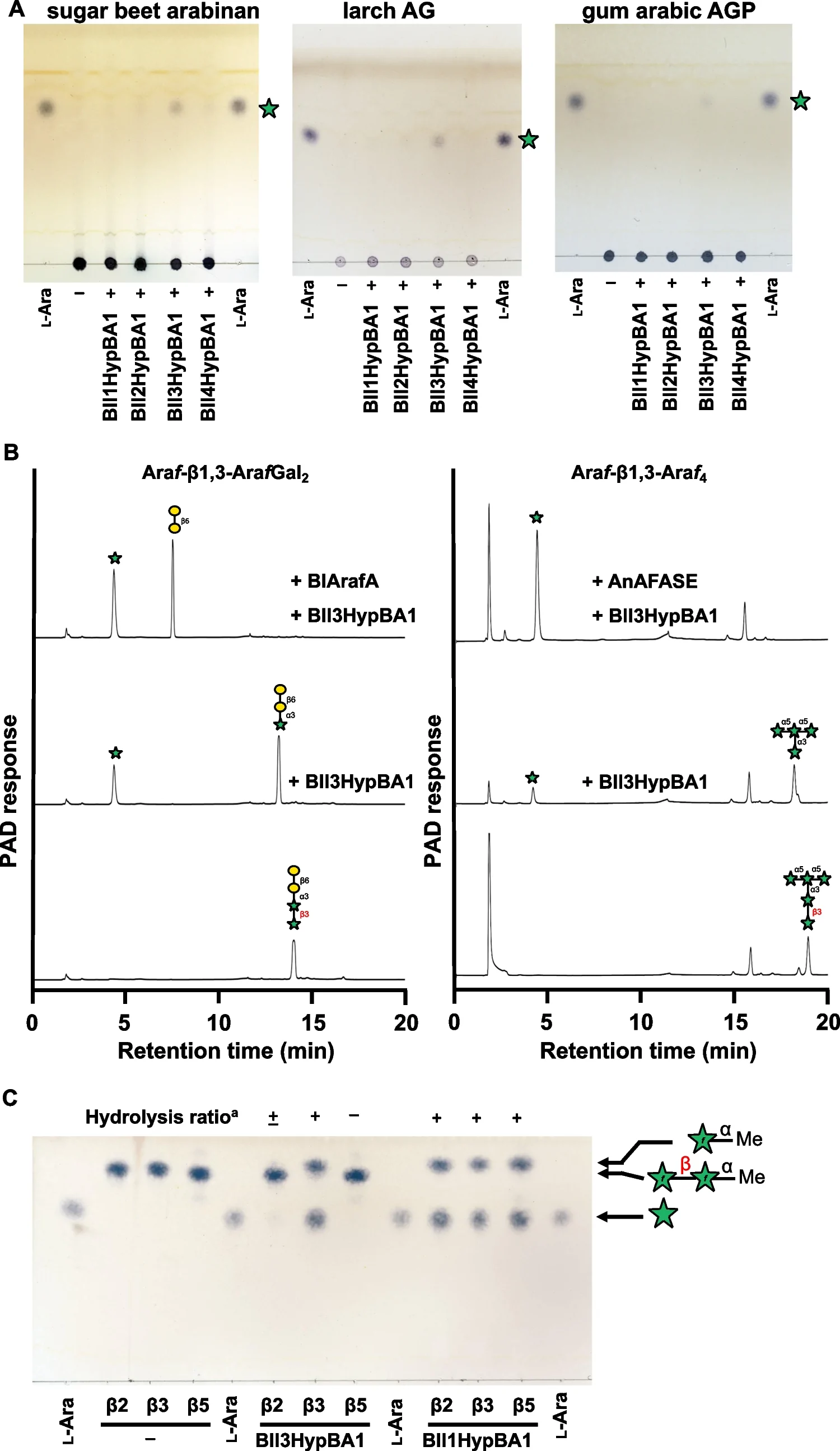
Substrate specificities of Bll3HypBA1. **A** TLC analysis using sugar beet arabinan, larch AG, and gum arabic AGP as substrates. The substrates were incubated in the absence ( −) or presence ( +) of enzymes (Bll1HypBA1, Bll2HypBA1, Bll3HypBA1-NΔ35CΔ761, or Bll4HypBA1) at 37 °C for 16 h.** B** HPAEC-PAD analysis using Ara*f*-β1,3-Ara*f*Gal_2_ and Ara*f*-β1,3-Ara*f*_4_ as substrates. The substrates were incubated with Bll3HypBA1-NΔ379CΔ761 at 40 °C for 16 h. The hydrolysate was further incubated with α-l-arabinofuranosidases BlArafA or AnAFASE. **C** TLC analysis using Ara*f*-β1,2-Ara*f*-α-OMe (β2), Ara*f*-β1,3-Ara*f*-α-OMe (β3), and Ara*f*-β1,5-Ara*f*-α-OMe (β5) in the absence ( −) or presence of Bll3HypBA1-NΔ379CΔ933 or Bll1HypBA1 at 37 °C for 16 h. ^a^The substrate was ( +) completely hydrolyzed, ( ±) partially hydrolyzed, and ( −) not hydrolyzed

The substrate specificity of Bll3HypBA1 was compared with that of the other three paralogs of *B. longum* subsp. *longum* JCM 1217 using some natural polysaccharides as substrates. As shown in Fig. 3A, Bll3HypBA1-NΔ35CΔ761 released l-arabinose from sugar beet arabinan, larch AG, and gum arabic AGP, whereas other paralogs in *B. longum* subsp. *longum* JCM 1217 could not. This result suggests that Bll3HypBA1 has a specific ability to degrade the terminal β-l-Ara*f* moieties of these polysaccharides. The l-arabinose content after the overnight reaction of Bll3HypBA1 was 0.32% for sugar beet arabinan, 0.39% for larch AG, and 0.20% for gum arabic AGP. This result indicates that a small amount of terminal β-l-Ara*f* residues is present in AGPs and arabinans. Bll3HypBA1 also released l-arabinose from Ara*f*-β1,3-Ara*f*Gal_2_ (Fig. 3B left). The mass of the degraded Ara*f*Gal_2_ was *m/z* 474.40 (calc. *m/z* 474.16), which was a reduction in the mass of l-arabinose (MW 132) from Ara*f*-β1,3-Ara*f*Gal_2_ (*m/z* 606.48; calc. *m/z* 606.20). Subsequently, the released Ara*f*Gal_2_ was further hydrolyzed to β1,6-Gal_2_ by α-l-arabinofuranosidase BlArafA. Bll3HypBA1 also released l-arabinose from Ara*f*-β1,3-Ara*f*_4_ (*m/z* 678.77; calc. *m/z* 678.22), and the hydrolysate Ara*f*_4_ was further degraded by *A. niger* α-l-arabinofuranosidase AnAFASE (Fig. 3B, right). The specific activity of Bll3HypBA1-NΔ379CΔ761 for Ara*f*-β1,3-Ara*f*Gal_2_ was two times higher than that for Ara*f*-β1,3-Ara*f*_4_ (Table 2). In a comparison of the specific activities of larch AG, sugar beet arabinan, and gum arabic AGP, Bll3HypBA1-NΔ35CΔ761 with N-terminal LamG was 5.8-, 1.7-, and 1.1-fold higher than Bll3HypBA1-NΔ379CΔ761 without N-terminal LamG, respectively (Table S2). Alternatively, Bll3HypBA1-NΔ35CΔ761 was 1.6-fold lower than Bll3HypBA1-NΔ379CΔ761 for Ara*f*-β1,3-Ara*f*Gal_2_.

**Table 2:** Substrate specificity of Bll3HypBA1 toward oligosaccharides containing terminal β-Ara*f* structure

Substrates	Conc. (mM)	Specific activity^a^ (units/mg)	Relative activity^b^ (%)
Ara*f-*β1,3-Ara*f*Gal_2_	0.15	13	100
Ara*f-*β1,3-Ara*f*_4_	0.15	6.9	51
Ara*f*-β-*p*NP	1.0	0.11	0.82
Ara*f-*β-OMe	0.40	0.0027	0.021
Ara*f*-β-Hyp(*cis*)	0.11	0.00027	< 0.01
Ara*f*-β-Hyp(*trans*)	0.11	0.0071	0.053
Ara*f*-β1,2-Ara	0.35	0.011	0.079
Ara*f*-β1,2-Ara*f*-β1,2-Ara*f*-β-Hyp	1.0	0.00011	< 0.01
Ara*f*-β1,2-Ara*f*-β-Hyp	0.13	0.00043	< 0.01

^a^Bll3HypBA1-NΔ379CΔ761 was used for the enzymatic reactions as described in Materials and Methods

^b^Relative activity was expressed as the percentage of the activity toward Ara*f-*β1,3-Ara*f*Gal_2_

Furthermore, synthetic arabinosyl disaccharides containing terminal β1,2-, β1,3-, and β1,5-Ara*f* structures were used for a detailed evaluation of the linkage specificity of this enzyme (Ishiwata et al. 2022a). Bll3HypBA1 released l-arabinose from Ara*f*-β1,3-Ara*f*-α-OMe and slightly from Ara*f*-β1,2-Ara*f*-α-OMe but not from Ara*f*-β1,5-Ara*f*-α-OMe (Fig. 3C). Conversely, Bll1HypBA1 completely hydrolyzed these substrates. Bll3HypBA1 also exhibited < 1% degradative activity for Ara*f*-β-*p*NP, Ara*f-*β-OMe, Ara*f*-β-Hyp glycosides, and β-AOSs containing Ara*f*-β1,2-linkage compared with Ara*f*-β1,3-Ara*f*Gal_2_ containing Ara*f*-β1,3-linkage (Table 2). These results showed that Bll3HypBA1 is a β-l-arabinofuranosidase specific for Ara*f*-β1,3-Ara*f* structures.

The optimal temperature and pH for Ara*f*-β1,3-Ara*f*Gal_2_-ABEE were 50 °C and 5.5, respectively (Fig. S3). Transglycosylation products were detected via TLC when 5% methanol, ethanol, and 1-propanol were used as acceptors (Fig. S4). These Rf values of TLC spots were similar to previous ones for Bll1HypBA1 (Fujita et al. 2014b). This result indicates that Bll3HypBA1 is the same anomer-retaining enzyme as other GH127/146 enzymes.

**Fig. 4:**
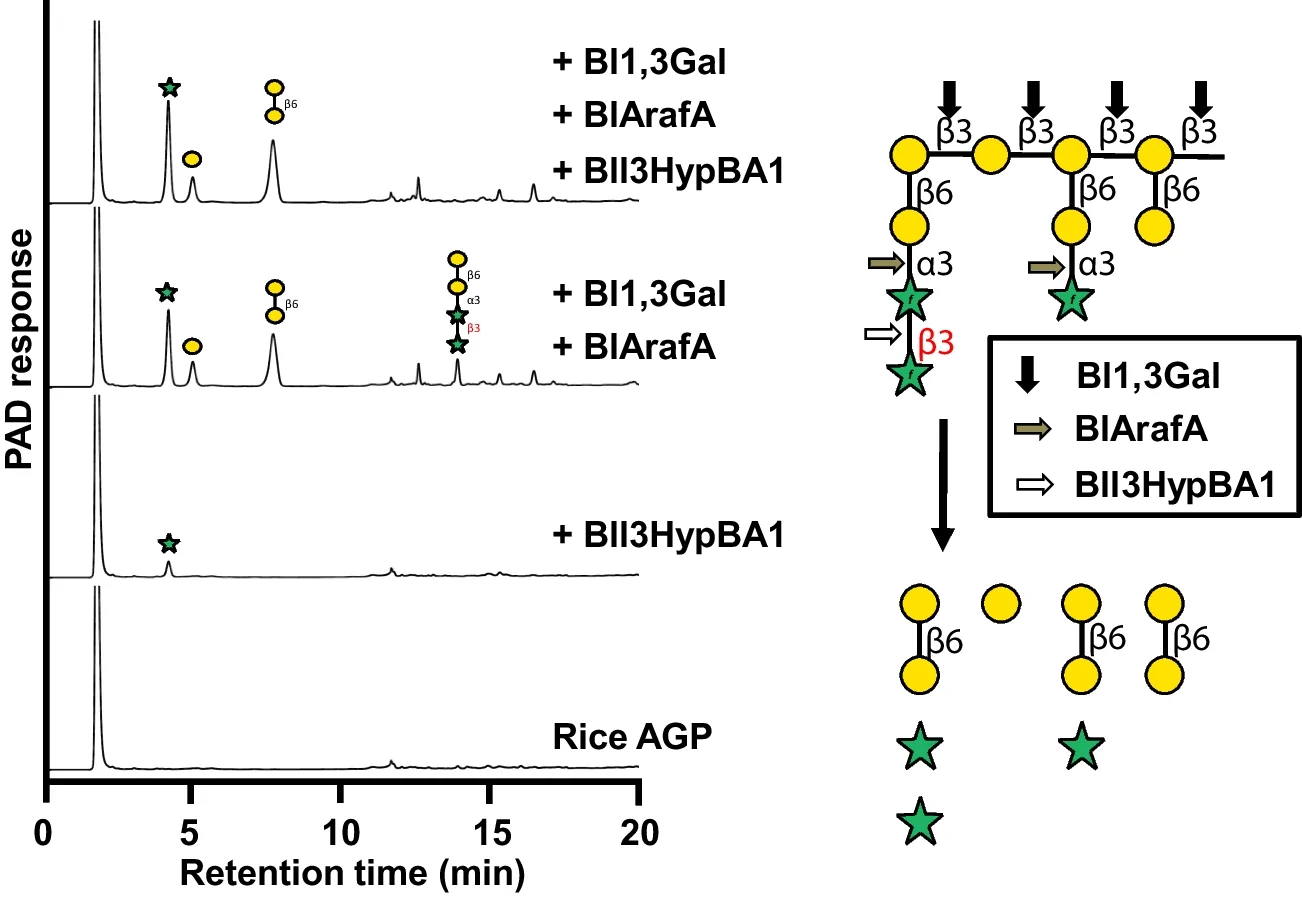
Stepwise hydrolysis of rice AGP by bifidobacterial AGP degradative enzymes. Rice AGP was incubated with a combination of Bl1,3Gal, BlArafA, and Bll3HypBA1-NΔ35CΔ761. A schematic drawing is shown in the right column. The cleavage sites of enzymes: Bl1,3Gal (black arrow), BlArafA (gray arrow), and Bll3HypBA1 (white arrow)

The rice anther AGP was predicted to contain Ara*f*-β1,3-Ara*f*Gal_2_ side chains, as depicted in the schematic in Fig. 4’s right column. To clarify the importance of Bll3HypBA1 in the degradation of AGPs, a combination reaction with AGP-degrading enzymes was performed. The reaction of Bll3HypBA1 alone produced a small amount of l-arabinose, whereas the reaction of Bl1,3Gal and BlArafA without Bll3HypBA1 produced Ara*f*-β1,3-Ara*f*Gal_2_ in addition to l-arabinose, galactose, and β1,6-Gal_2_ (Fig. 4). In contrast, Ara*f*-β1,3-Ara*f*Gal_2_ was completely degraded to l-arabinose and β1,6-Gal_2_ in a combination reaction involving three enzymes. The releasing β1,6-Gal_2_ is probably degraded to galactose by intracellular GH42 β-galactosidase (BLLJ_0443) in *B. longum* subsp. *longum* JCM 1217 (Fujita et al. 2019b). These findings suggest that Bll3HypBA1 is an effective cell surface anchoring enzyme for the degradation of AGPs.

### Crystal structure of Bll3HypBA1

The crystal structure of Bll3HypBA1-NΔ379CΔ761 (aa: 380–1223) was determined at a resolution of 1.75 Å by the single-wavelength anomalous dispersion using a SeMet-derivative (Table S3). The NΔ379CΔ761 structure contained one molecule per asymmetric unit, and residues M379–V1051 were modeled. In the active site of the NΔ379CΔ761 structure, a Zn atom coordinated by E723, C725, C804, and C805 was clearly observed, and a Tris molecule was bound to the l-arabinose-binding site (subsite − 1) (Fig. S5A and B). A crystallographic anomalous scattering analysis was performed to identify the metal atom (Table S3). A Bijvoet difference density map of the data collected at 1.280 Å wavelength revealed a prominent peak at the metal site, whereas a difference map of the data collected at 1.300 Å wavelength, which is above the absorption edge of Zn (1.2837 Å), showed a significant decrease in the anomalous scattering ability (Fig. S5C). This result confirmed that the metal ion bound at the active site is Zn^2+^.

Since the region following residue 1052 in the NΔ379CΔ761 structure was disordered, we also crystallized the Bll3HypBA1-NΔ379CΔ933 (aa: 380–1051) construct. The crystal structure of NΔ379CΔ933 was determined at a resolution of 1.70 Å (Table S3). The NΔ379CΔ933 structure contained two molecules per asymmetric unit, and residues from M379 to E1050 were modeled for both chains (A and B). The three molecules, including one in NΔ379CΔ761 and two in NΔ379CΔ933, had almost identical main chain structures, as the root mean square deviation (RMSD) values for the Cα atoms between all chain pairs were less than 0.24 Å. The Zn-coordination site structure of NΔ379CΔ933 was also almost identical to that of NΔ379CΔ761 (Fig. S5D), but no Tris molecule was identified in the active site. Therefore, we primarily focused on the chain A structure of NΔ379CΔ933.

**Fig. 5:**
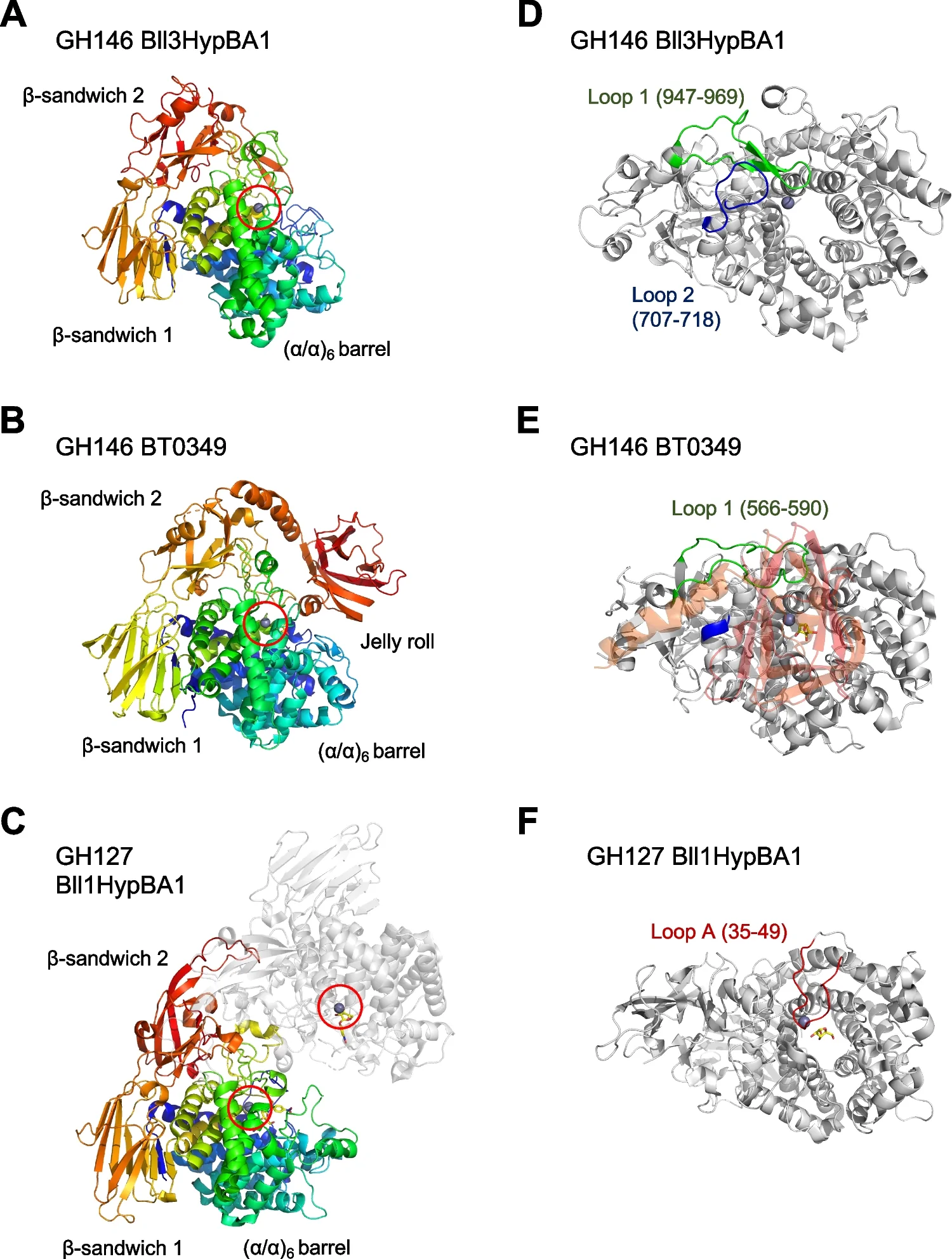
Overall and the catalytic domain structures of the GH146 and GH127 enzymes. **A**–**C** Domain structures of **A** GH146 Bll3HypBA1 and **B** GH146 BT0349 and dimer structure of **C** GH127 Bll1HypBA1. A ribbon presentation with a rainbow color (blue to red from the N- to C-terminus) is shown. Red circles indicate the Zn atoms at the active site (gray spheres). **D**–**F** Top views of the catalytic domain of **D** GH146 Bll3HypBA1, **E** GH146 BT0349, and **F** GH127 Bll1HypBA1. In **E**, an α-helix linker and the C-terminal jelly roll domain are shown in transparent color (orange to red)

The crystal structure of GH146 Bll3HypBA1 consists of three domains: catalytic (α/α)_6_ barrel (aa: 379–820), β-sandwich 1 (aa: 821–920), and β-sandwich 2 (aa: 921–1051) (Fig. 5A). The GH146 family member BT0349 has an α-helical linker and a jelly roll domain following the β-sandwich 2 domain (Fig. 5B). Although the C-terminal jelly roll domain of BT0349 is structurally similar to carbohydrate-binding module (CBM) family 35, the function of this domain remains unknown (Luis et al. 2018). While the GH146 enzymes (Bll3HypBA1 and BT0349) are monomeric in solution, GH127 Bll1HypBA1 forms a homodimer by interactions between the β-sandwich 2 domains (Fig. 5C). Extended structural elements in the β-sandwich 2 domain of the GH146 enzymes (a loop with two β-strands in Bll3HypBA1 and the jelly roll domain in BT0349) interact with the catalytic domain (Fig. 5A and B).

Figure 5D, E, and F depict the top views of the catalytic domain of GH146 and GH127 enzymes. In Bll3HypBA1, loop 1 in β-sandwich 2 (aa: 947–969) partially covers the active site, whereas loop 2 (aa: 707–718) supports loop 1 (Fig. 5D). The C-terminal jelly roll domain of BT0349 shields its active site (Fig. 5E, transparent red). Loop 1 (aa: 566–590) is distant from the active site in BT0349, whereas loop 2 is absent. In GH127 Bll1HypBA1, the active site is covered by a long loop A (aa: 35–49) in the (α/α)_6_ barrel domain (Fig. 5F) (Ito et al. 2014).

**Fig. 6:**
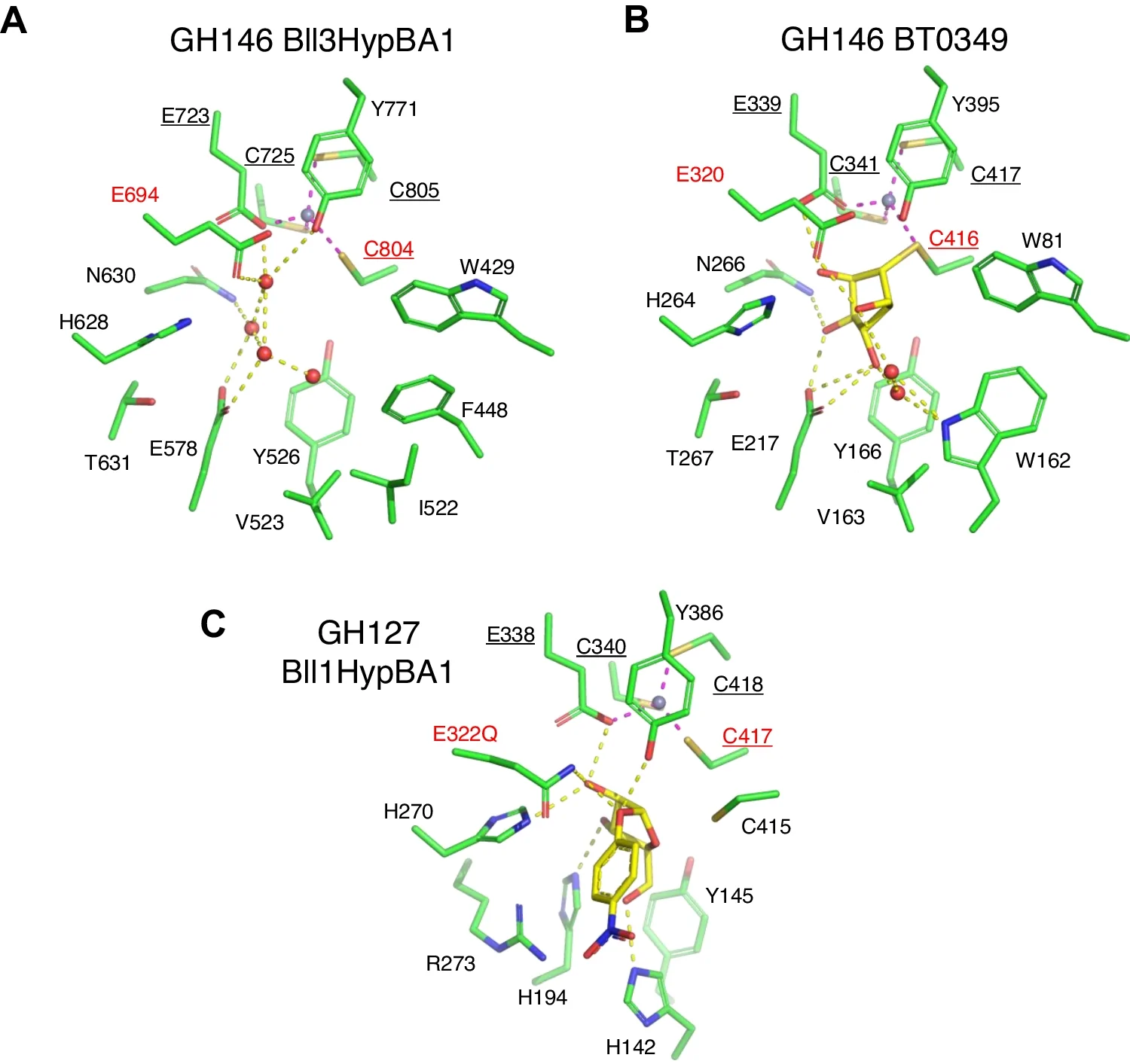
Active site structures of the GH146 and GH127 enzymes. **A** GH146 Bll3HypBA1, **B** GH146 BT0349, and **C** GH127 Bll1HypBA1. The label characters of the Zn-coordinating residues are underlined. The catalytic residues (nucleophile and acid/base catalyst) are indicated by labels of red characters

Figure 6 depicts the active site structures of the GH146 and GH127 enzymes. The Zn atom is coordinated by E723, C725, C804, and C805 in Bll3HypBA1 (Fig. 6A). The Zn-coordination structure of Bll3HypBA1 is identical to that of BT0349 and Bll1HypBA1 (Fig. 6B and C). R273, H194, H142, and Y145 form the substrate binding site of GH127 Bll1HypBA1 (Fig. 6C), whereas these residues are completely different in GH146 enzymes. F448 and I522 occupy the active site in Bll3HypBA1 (Fig. 6A), whereas W162 occupies this position in BT0349 (Fig. 6B). Therefore, these residues may determine the β-linkage specificity of Bll3HypBA1.

### Mutational analysis

**Fig. 7:**
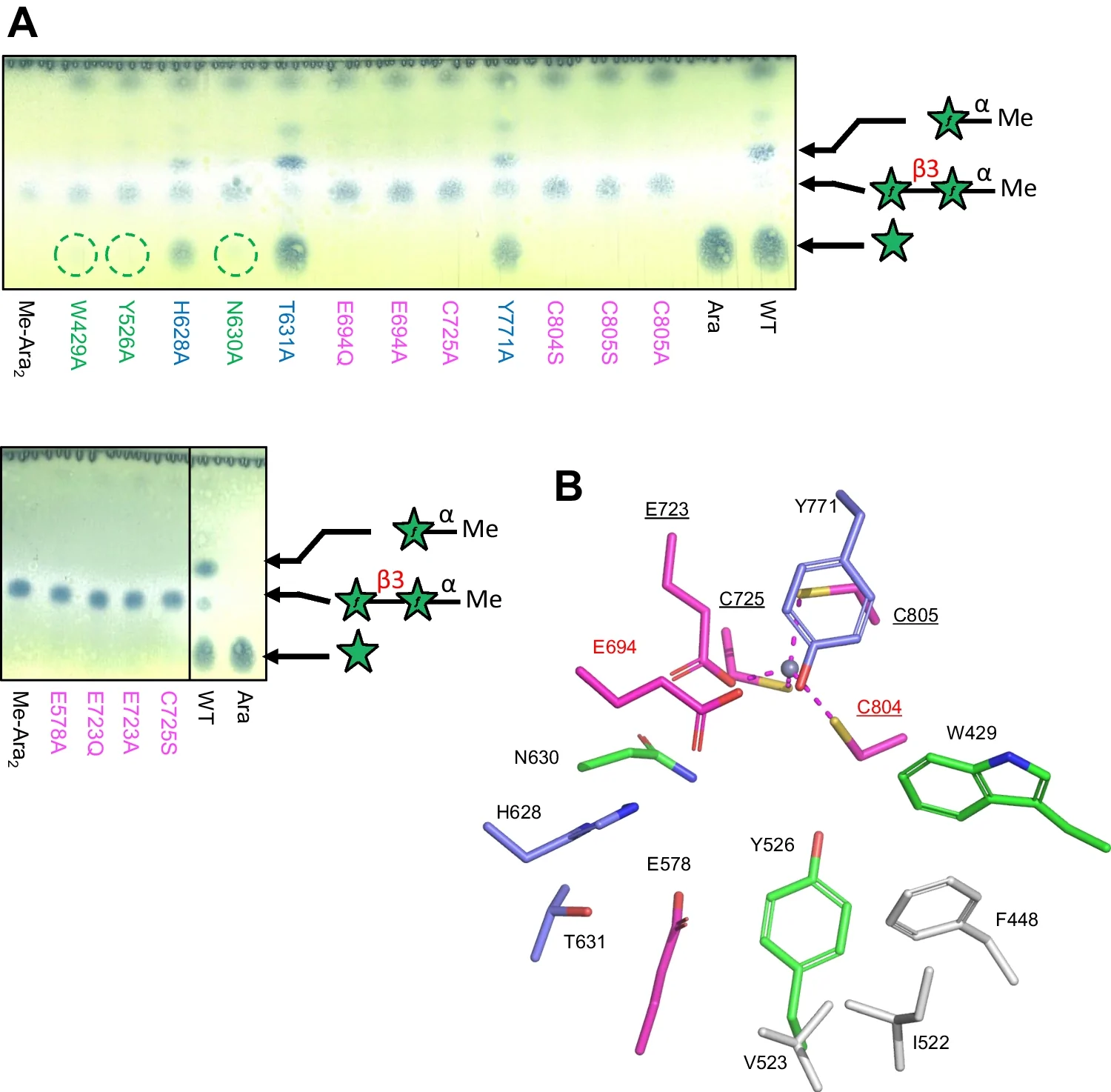
Mutational analysis of Bll3HypBA1. **A** TLC analysis of the wild-type Bll3HypBA1-NΔ379CΔ933 and its mutants using Ara*f*-β1,3-Ara*f*-α-OMe at 37 °C for 30 min. **B** Active site residues colored by the effects of mutation: magenta, no activity detected for mutants; green, weak activity detected by TLC; blue, activity detected; white, not examined

Next, using site-directed mutagenesis, we determined the significance of active site residues on enzyme activity (Fig. 7). Substitutions were made at the Zn-coordinating residues (E723, C725, C804, and C805), catalytic residues (E694 and C804), and residues near the catalytic center (W429, Y526, E578, H628, N630, T631, and Y771). The activity of the mutants was analyzed via TLC using Ara*f*-β1,3-Ara*f*-α-OMe as the substrate. The mutant enzymes at the Zn-coordinating and catalytic residues (E694Q, E694A, E723Q, E723A, C725S, C725A, C804S, C805S, and C805A) completely lost their activity (Fig. 7A). HPLC analysis also confirmed the importance of these residues (Table S4). For other active site residues, E578A also lost its activity, whereas W429A, Y526A, and N630A exhibited slight spots of l-arabinose release on TLC, indicating significantly diminished activity (Fig. 7A). H628A, T631A, and Y771A exhibited residual spots of the substrate Ara*f*-β1,3-Ara*f*-α-OMe but clear spots of l-arabinose after reaction at 37 °C for 30 min, indicating that these mutants retained their activity.

The result of the mutational analysis is shown in Fig. 7B by color codes. Residues without activity, residues with slight activity, and residues with significant activity in the mutant enzymes are depicted in magenta, green, and blue, respectively. The Zn-coordinating three Cys and one Glu residue and the acid/base catalyst residue were essential for activity. In the active site, E578 mutants lacked activity. W429 and E578 may participate in substrate recognition at subsite − 1 (Fig. 6A). N630 and Y526 play significant roles in the activity as well. H628, T631, and Y771 are involved in substrate recognition but are not required for activity. These nonessential residues may recognize the reducing end side of Ara*f*-β1,3-Ara*f* at subsite + 1.

## Discussion

Previously, we characterized AGP-degrading enzymes in *B. longum* subsp. *longum.* The degradative enzymes are encoded by an AGP degradation gene cluster containing GH43_24 Bl1,3Gal (BLLJ_1840) and GH30_5 Bl1,6Gal (BLLJ_1841) and a GH43 gene cluster containing five GH43 α-l-arabinofuranosidases. GH43_22 BlArafA (BLLJ_1854) acts on the α1,3-linked Ara*f* of AGP (Fujita et al. 2019a), GH43_22 BlArafB (BLLJ_1853) acts on the α1,5-linked Ara*f* of the arabinan backbone, and GH43_27 BlArafC (BLLJ_1852) acts on the α1,2- and α1,3-linked Ara*f* on the arabinan side chains (Komeno et al. 2019). Recently, BlArafD (BLLJ_1851) and BlArafE (BLLJ_1850) with tandem GH43 domains have been characterized. The GH43_UC (uncharacterized subfamily of GH43) domain of BlArafD acts on the α1,2-linked Ara*f* of the α1,2- and α1,3-Ara*f* doubly substituted arabinoxylan, whereas the GH43_26 BlArafD domain acts on the arabinan backbone (Komeno et al. 2022). In contrast, the GH43_22 BlArafE acts on the α1,3-linked Ara*f* of the α1,3- and α1,4-Ara*f* doubly substituted gum arabic AGP, whereas GH43_34 BlArafE acts on the remaining α1,4- linked Ara*f* (Sasaki et al. 2022). A mixture of Bl1,3Gal, Bl1,6Gal, and BlArafA had a synergistic effect on the degradation of larch AG but only released 3.3% of the polysaccharide’s sugar component (Fujita et al. 2019a). A GH127 β-l-arabinofuranosidase, BT3674, in *Bacteroides thetaiotaomicron* showed a synergistic effect with exo-β-1,3-galactanases for the degradation of larch AG (Cartmell et al. 2018). Notably, Bll3HypBA1 (BLLJ_1848) is flanked by the AGP degradation gene cluster and GH43 gene cluster. In addition to Bll3HypBA1, all AGP degradation enzymes and GH43 α-l-arabinofuranosidases contain C-terminal membrane anchoring regions, indicating that these enzymes act synergistically to degrade AGPs and arabinans on the bifidobacterial cell surface. In contrast, the AGP degradation gene cluster is conserved in nearly all *B. longum* subsp. *longum* strains, whereas the Bll3HypBA1 ortholog (> 99% identity) was conserved in only 31 strains (5.2%) in 600 *B. longum* and *B. longum* subsp. *longum* strains in the NCBI genome database. In addition, the orthologous proteins (> 78% identity) are not conserved in other bifidobacterial species except for strains of *B. aesculapii* and *B. primatium*, which were isolated from the feces of common marmosets and cotton-top tamarins, respectively. These findings indicate that the Bll3HypBA1 ortholog is not a universal gene in bifidobacteria.

Bll3HypBA1 contains two independent LamG domain modules. Upon comparing the reactivities of Bll3HypBA1-NΔ35CΔ761 with N-terminal LamG and Bll3HypBA1-NΔ379CΔ761 without LamG, the former were more reactive for polysaccharides than the latter (Table S2). Notably, the N-terminal LamG domain was conserved in two adjacent AGP degradative enzymes (Bl1,3Gal and Bl1,6Gal) and four GH43 α-l-arabinofuranosidases (BlArafB-E) in *B. longum* subsp. *longum* JCM 1217. CBMs in bifidobacteria are predicted to serve as substrate and/or hydrolysate docking stations (van den Broek et al. 2008). The N-terminal LamG domain may aid in the degradation of AGPs and arabinans for bifidobacterial cell surface anchoring enzymes.

In the present study, the crystal structure of Bll3HypBA1 was determined as the second three-dimensional structure of the GH146 member following BT0349. BT0349 has a C-terminal jelly roll domain that covers the active site, and this enzyme does not appear to bind polymer substrates (Fig. 5B). BT0349 is present in the PUL of rhamnogalacturonan-I (RG-I), which comprises multiple GHs and is anticipated to act on oligosaccharides cleaved by other GHs. In contrast, Bll3HypBA1 can act independently on rice AGP (Fig. 4). This is consistent with the structural characteristics of Bll3HypBA1, which contains no large structural element covering the active site. The Zn atom of Bll3HypBA1 is coordinated by Cys_3_-Glu residues, and GH127 and GH146 enzymes share this structural characteristic (Cartmell et al. 2018; Ito et al. 2014; Luis et al. 2018; Maruyama et al. 2022; McGregor et al. 2021). In our previous studies, we elucidated the reaction mechanism of GH127 Bll1HypBA1 using synthetic inhibitors that were specifically designed for cysteine glycosidase (Ishiwata et al. 2022b; Maruyama et al. 2022). It has been demonstrated that the coordination of cysteine to Zn^2+^ is necessary for deglycosylation from the thioglycosyl intermediate, which is energetically unfavorable in the absence of metal coordination (McGregor et al. 2021). Consequently, this zinc coordination structure is shared by clan GH-P (GH127 and GH146) with cysteine as the catalytic nucleophilic residue. In addition, we performed a mutational analysis of the active site residues of Bll3HypBA1 and demonstrated the significance of these residues for the enzyme activity toward β1,3-linked Ara*f* disaccharide. In addition to Zn-coordinating and catalytic residues, E578 was demonstrated to be essential for activity. Several other active site residues have been shown to contribute significantly to the enzyme activity, providing a structural basis for the strict recognition toward the Ara*f*-β1,3-linkages.

Although it is known that gum arabic AGP from *Acacia senegal* is partially substituted by Gal-α1,3- or Ara*p*-β1,3- at the Ara*f*-α1,3-terminal sugar (Sasaki et al. 2021), the presence of Ara*f*-β1,3 substitution has never been reported. Bifidobacterial GH39 3-*O*-α-d-galactosyl-α-l-arabinofuranosidase has been previously characterized for the release of Gal-α1,3-Ara*f* disaccharide and 3-*O*-β-l-arabinopyranosyl-α-l-arabinofuranosidase for the release of Ara*p*-β1,3-Ara*f* disaccharide on AGP (Sasaki et al. 2021, 2023). The presence of β-Ara*f* in gum arabic AGP and larch AG has been demonstrated, but the linkage positions remain unclear (Cartmell et al. 2018). In addition to quinoa arabinan (Wefers et al. 2014) and rice AGP (Kawaguchi et al. 1996), we found Ara*f*-β1,3 substitution in larch AG, gum arabic AGP, and sugar beet arabinan. These results indicated that AGPs and arabinans may contain terminal Ara*f*-β1,3-linkages. Since partially substituted sugars prevent enzymatic degradation of these polysaccharides by bacteria, the presence of Bll3HypBA1 and orthologous enzymes is advantageous for some *Bifidobacterium* and *Bacteroides* species with AGP-degrading ability. Jones et al. found GH39 α-l-(β-1,2)-arabinofuranobiosidase from rumen fungi; this enzyme releases Ara*f*-β1,2-Ara*f* disaccharide on sugar beet arabinan (Jones et al. 2017). To the best of our knowledge, Ara*f*-β1,3-Ara*f* disaccharide-releasing enzyme has not been discovered. Bll3HypBA1 is useful for glycan structure analysis, oligosaccharide preparation, and Ara*f*-β1,3 content measurement due to its specificity for the Ara*f*-β1,3-Ara*f* structure.

## Supplementary Information

Below is the link to the electronic supplementary material.

Supplementary file1 (PDF 1628 KB)

## Data Availability

The datasets generated during and/or analyzed during the current study are available from the corresponding author on reasonable request.
