# Supplementary material for: Bifidobacterial GH146 β-l-arabinofuranosidase for the removal of β1,3-l-arabinofuranosides on plant glycans
Source: Appl Microbiol Biotechnol. 2024 Feb 7;108:199. doi: 10.1007/s00253-024-13014-8 (PMC10850190; doi:10.1007/s00253-024-13014-8)
Supplement: Supplementary file 1 [file 253_2024_13014_MOESM1_ESM.pdf]

Bifidobacterial GH146 β-L-arabinofuranosidase for the removal of β1,3-L-arabinofuranosides on plant glycans

Kiyotaka Fujita[1,2]*, Hanako Tsunomachi[1], Pan Lixia[3,4], Shun Maruyama[3], Masayuki Miyake[3], Aimi Dakeshita[1], Kanefumi Kitahara[1,2], Katsunori Tanaka[5,6], Yukishige Ito[5,7], Akihiro Ishiwata[5], Shinya Fushinobu[3,8]*

1. Faculty of Agriculture, Kagoshima University, 1-21-24 Korimoto, Kagoshima, Kagoshima 890-0065, Japan

2. The United Graduate School of Agricultural Sciences, Kagoshima University, 1-21-24 Korimoto, Kagoshima, Kagoshima 890-0065, Japan

3. Department of Biotechnology, The University of Tokyo, 1-1-1 Yayoi, Bunkyo-ku, Tokyo 113-8657, Japan

4. The National Engineering Research Center for Non-Food Biorefinery, State Key Laboratory of Non-Food Biomass and Enzyme Technology, Guangxi Academy of Sciences, Nanning 530007, Guangxi, China

5. RIKEN, Cluster for Pioneering Research, 2-1 Hirosawa, Wako, Saitama 351-0198, Japan

6. Department of Chemical Science and Engineering, Tokyo Institute of Technology
2-12-1 Ookayama, Meguro-ku, Tokyo 152-8552, Japan

7. Graduate School of Science, Osaka University
1-1 Machikaneyama-cho, Toyonaka, Osaka 560-0043 Japan

8. Collaborative Research Institute for Innovative Microbiology, The University of Tokyo, 1-1-1 Yayoi, Bunkyo-ku, Tokyo, 113-8657, Japan

* **Address correspondence to:** Kiyotaka Fujita, k4022897@kadai.jp; Shinya Fushinobu, asfushi@mail.ecc.u-tokyo.ac.jp

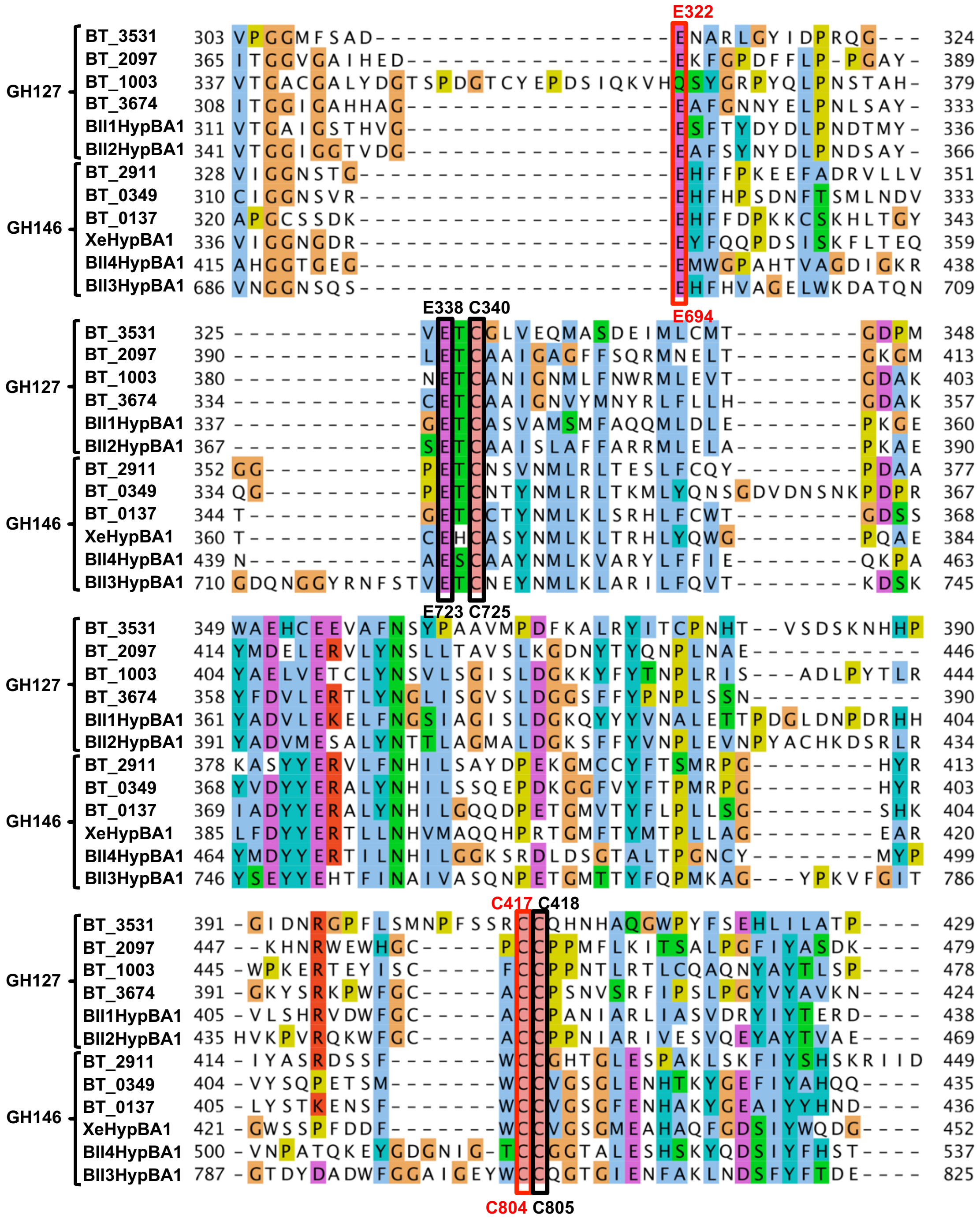

Figure S1. Amino acid sequence alignment of Bll3HypBA1 and GH127/146 β-L-arabinofuranosidases. The catalytic residues (nucleophile and acid/base catalyst) are boxed with magenta lines. The Zn-coordinating residues are boxed with black lines. The amino acid residues for Bll1HypBA1 and Bll3HypBA1 are shown above and below the box, respectively.

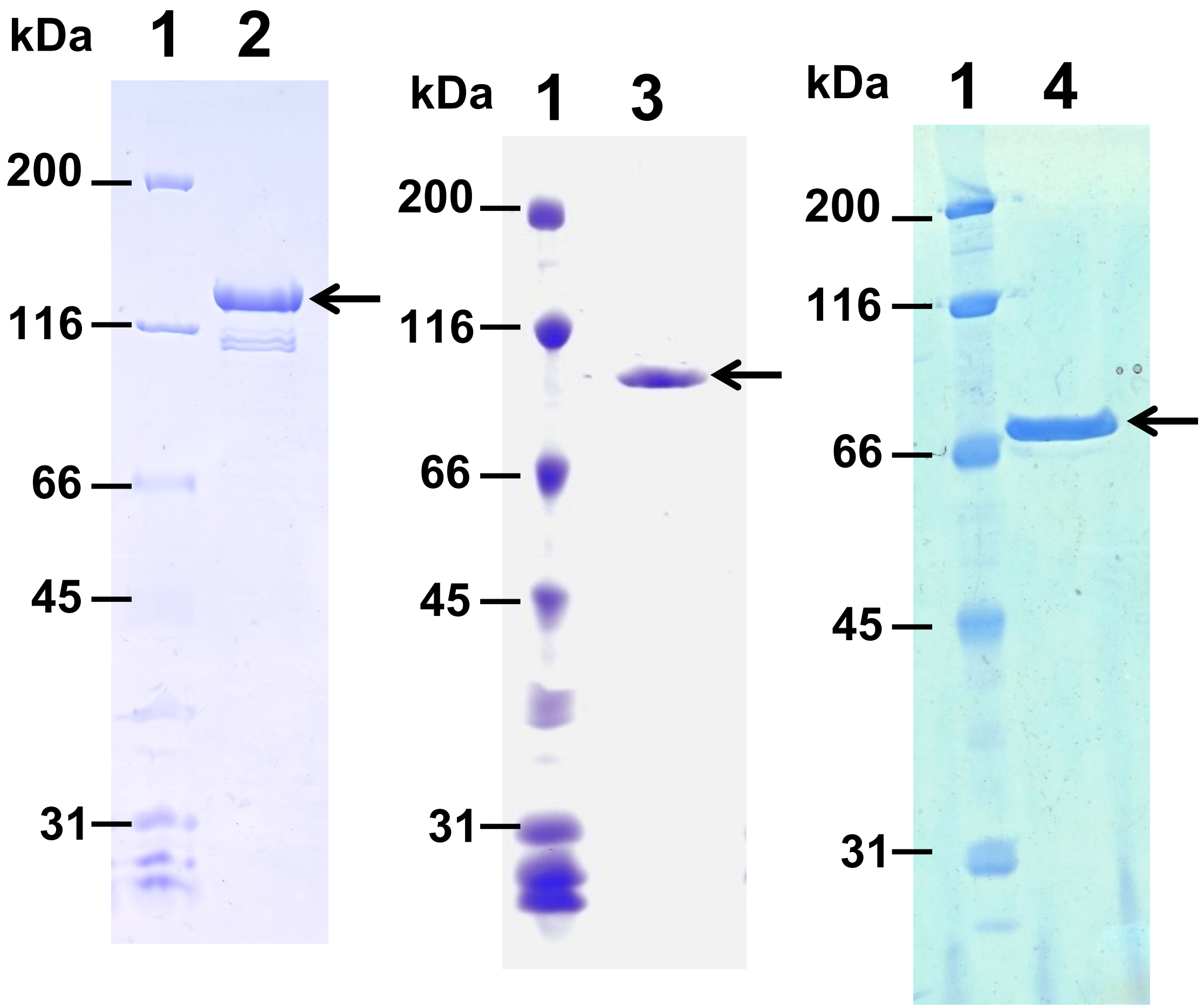

Figure S2. SDS-PAGE analysis of recombinant Bll3HypBA1 proteins. Purified proteins were electrophoresed on a 5–20% gradient polyacrylamide gel and stained with Coomassie Brilliant Blue R-250. Lane 1, molecular size marker; lane 2, Bll3HypBA1-NΔ35CΔ761; lane 3, Bll3HypBA1-NΔ379CΔ761; lane 4, NΔ379CΔ933 (lane 4). Arrows indicate target proteins at expected molecular size.

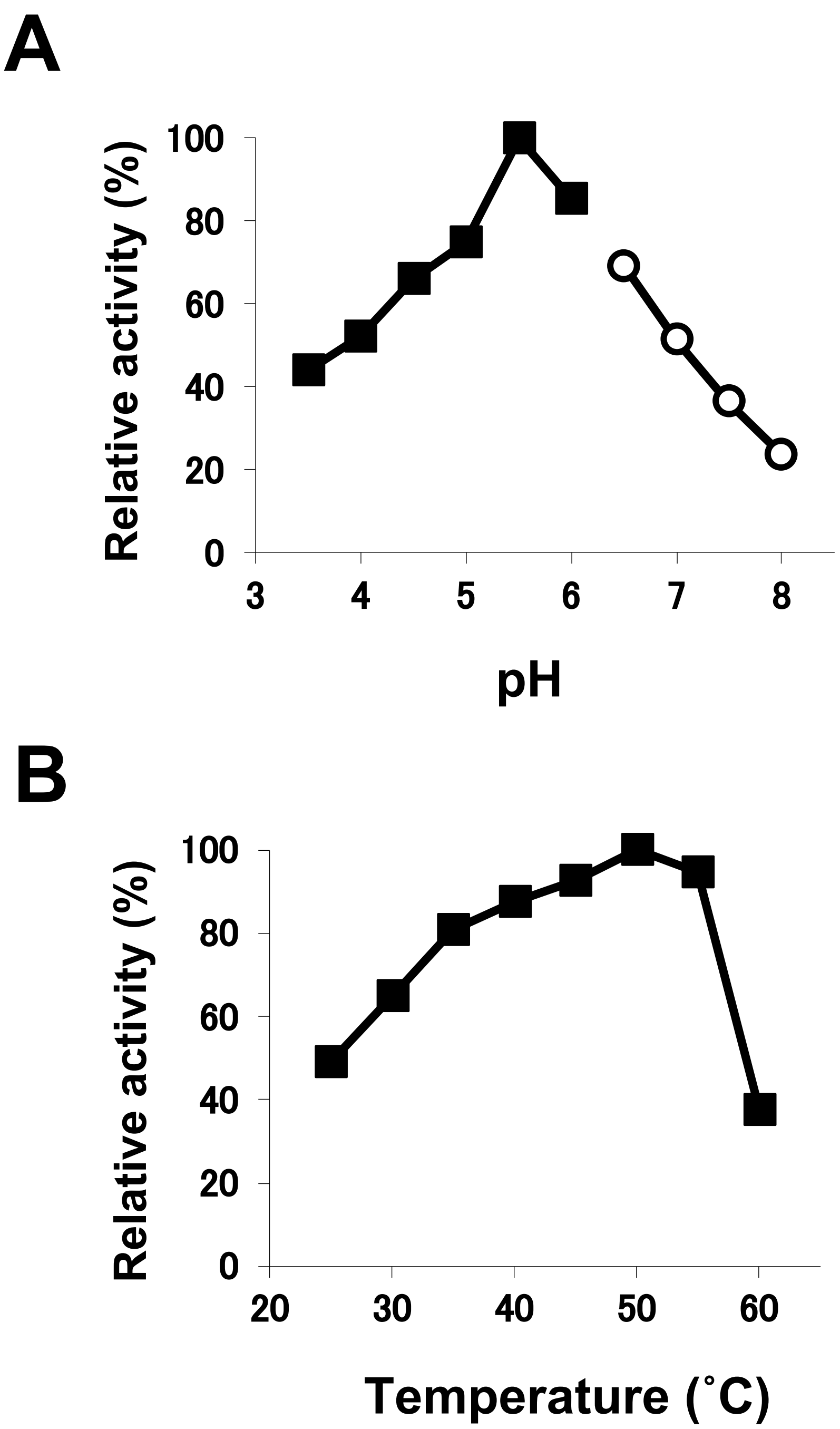

Figure S3. Optimal pH and temperature of Bll3HypBA1-NΔ35CΔ761. (**A**) pH dependence of the activity in various buffers at 40 °C for 20 min. Sodium acetate buffer (closed squares) and sodium phosphate buffer (open circles) were used. Enzyme activities are expressed as a percentage of the activity in sodium acetate buffer at pH 5.5. (**B**) The temperature dependence of the activity at pH 5.5 for 20 min. The enzymatic activities are expressed as the percentage of the activity at 50 °C.

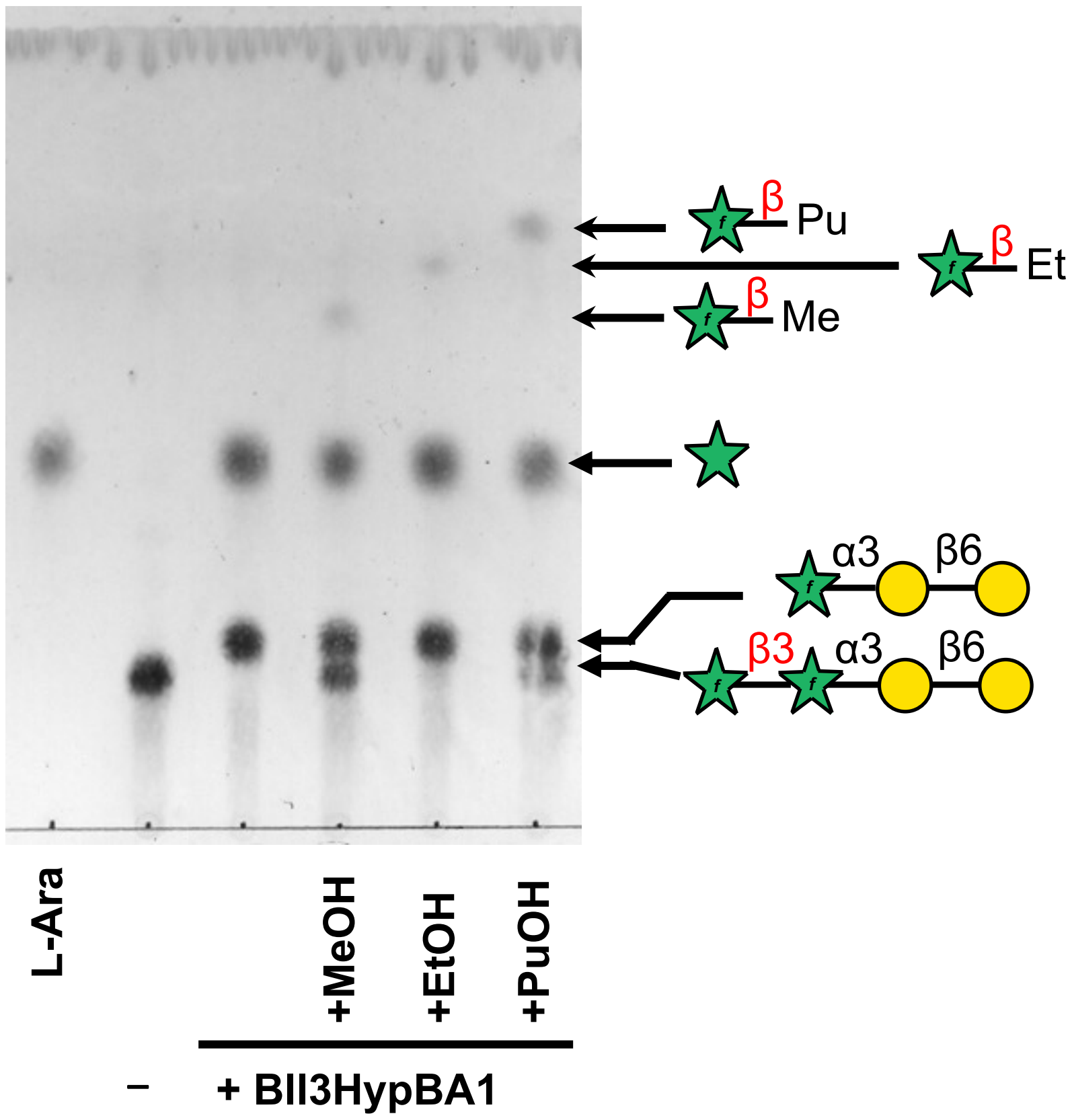

Figure S4. Transglycosylation activity of Bll3HypBA1 in the presence of 1-alkanols. Thin-layer chromatography analysis of reaction products. Bll3HypBA1-NΔ35CΔ761was incubated with L-Ara*f*-β1,3-L-Ara*f*-α1,3-Gal-β1,6-Gal in the presence of 5% methanol, ethanol, or 1-propanol.

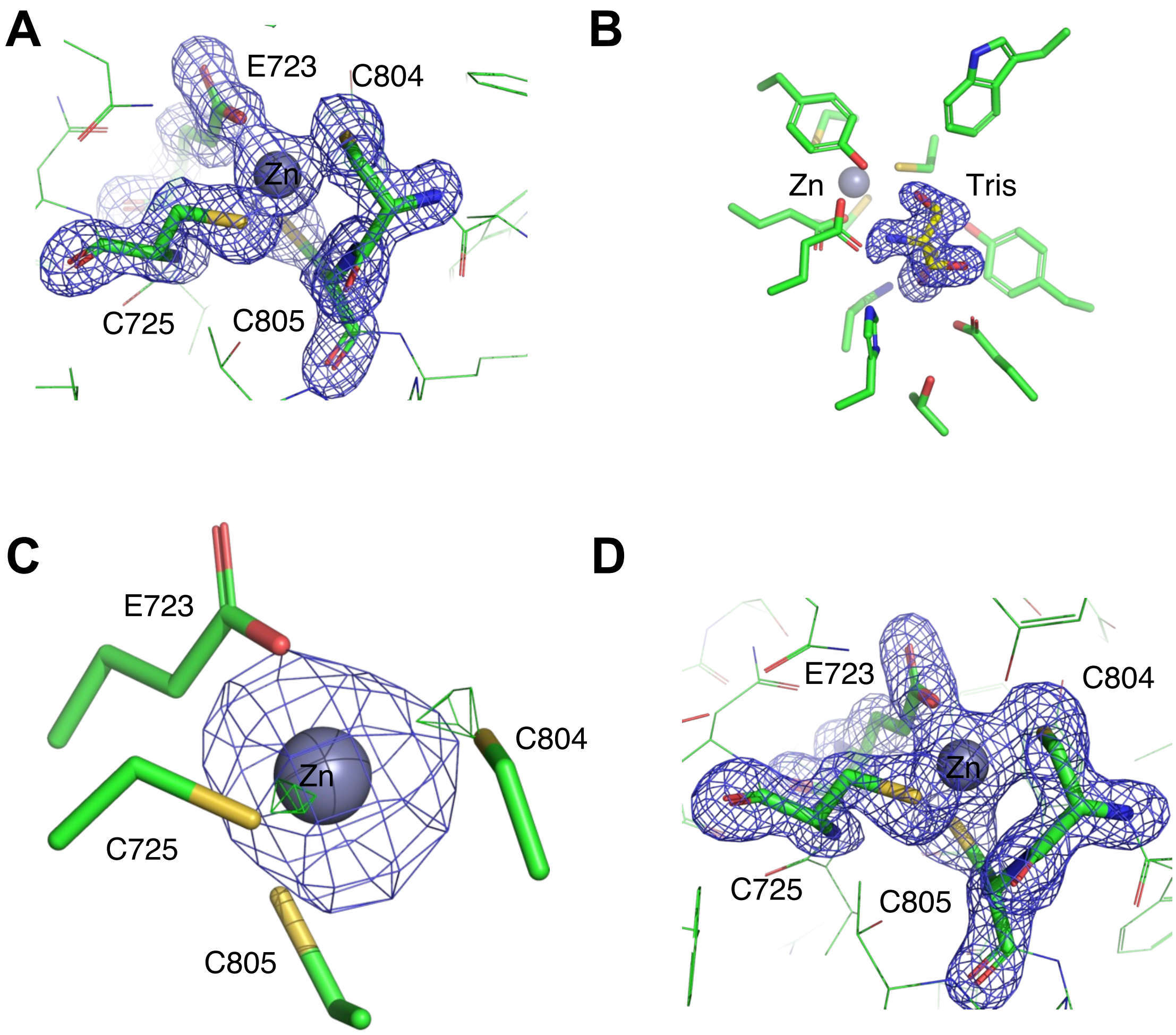

Figure S5. Electron density maps.

(**A**) Zn and coordinating residues in NΔ379CΔ761 with a polder map (3σ). (**B**) Tris molecule bound to the active site of NΔ379CΔ761 with a polder map (4σ). (**C**) Anomalous difference maps of the Zn atom. Data collected at 1.280 Å (5σ, blue) and 1.3000 Å (3.5σ, green) are shown. (**D**) Zn and coordinating residues in NΔ379CΔ933 with a polder map (3σ).

Table S1. The primers for the deletion and site-directed mutagenesis.

Name	Sequence of oligonucleotide primers
NΔ_Reverse_Primer	5´- CATGGTATATCTCCTTCTTAAAGTT -3´
NΔ379rev	5'- GAGAACGTCACCGTAGCCGACGAATA -3'
CΔ_Forward_Primer	5´-CTCGAGCACCACCACCACCACTG-3´
CΔ933rev	5'- GACCTCGGCCATATCCCAGTAGATC -3'
W429A mutation _for	5′- GGCGGTGCGGAGAACGGCCCGGACGAG-3′
W429A mutation _rev	5′- GTTCTCCGCACCGCCGTAGTTCTTCGC-3′
Y526A mutation _for	5′- CGCTTCGCGAACCTGCACAAGGTTGAG-3′
Y526A mutation _rev	5′- CAGGTTCGCGAACGGCACGATCAGGCC-3′
E578A mutation _for	5′- CGCACTGCGTACGGCGGCATGAATGAC-3′
E578A mutation _rev	5′- GCCGTACGCAGTGCGCAGCATGTCGGT-3′
H628A mutation _for	5′- GGCTTGGCGGCCAACACCACGATTCCG-3′
H628A mutation _rev	5′- GTTGGCCGCCAAGCCGTTGAGCGGATC-3′
N630A mutation _for	5′- CACGCCGCGACCACGATTCCGAAGCTC-3′
N630A mutation _rev	5′- CGTGGTCGCGGCGTGCAAGCCGTTGAG-3′
T631A mutation _for	5′- GCCAACGCGACGATTCCGAAGCTCACC-3′
T631A mutation _rev	5′- AATCGTCGCGTTGGCGTGCAAGCCGTT-3′
E694Q mutation _for	5′- CAGTCCCAGCACTTCCACGTGGCCGGT -3′
E694Q mutation _rev	5′- GAAGTGCTGGGACTGCGAGTTGCCGCC -3′
E694A mutation _for	5′- CAGTCCGCGCACTTCCACGTGGCCGGT -3′
E694A mutation _rev	5′- GAAGTGCGCGGACTGCGAGTTGCCGCC -3′
E723Q mutation _for	5′- ACCGTGCAGACCTGCAACGAGTACAAC -3′
E723Q mutation _rev	5′- GCAGGTCTGCACGGTGGAGAAGTTGCG -3′
E723A mutation _for	5′- ACCGTGGCGACCTGCAACGAGTACAAC -3′
E723A mutation _rev	5′- GCAGGTCGCCACGGTGGAGAAGTTGCG -3′
C725S mutation _for	5′- GAGACCAGCAACGAGTACAACATGCTC -3′
C725S mutation _rev	5′- CTCGTTGCTGGTCTCCACGGTGGAGAA -3′
C725A mutation _for	5′- GAGACCGCGAACGAGTACAACATGCTC -3′
C725A mutation _rev	5′- CTCGTTCGCGGTCTCCACGGTGGAGAA -3′
C804S mutation _for	5′- TATTGGAGCTGCCAGGGTACCGGTATC -3′
C804S mutation _rev	5′- CTGGCAGCTCCAATACTCGCCAATCGC -3′

C805S mutation _for	5′- TGGTGCAGCCAGGGTACCGGTATCGAG -3′
C805S mutation _rev	5′- ACCCTGGCTGCACCAATACTCGCCAAT -3′
C805A mutation _for	5′- TGGTGCGCGCAGGGTACCGGTATCGAG -3′
C805A mutation _rev	5′- ACCCTGCGCGCACCAATACTCGCCAAT -3′

The positions of the mutated sequences are underlined.

Table S2. Comparison of the specific activities of Bll3HypBA1-NΔ35CΔ761 and Bll3HypBA1-NΔ379CΔ761 towards oligosaccharide and polysaccharide.

Substrates	NΔ35CΔ761 (Units/μmol)[a]	NΔ379CΔ761 (Units/μmol)[a]	Fold[b]
Ara*f*-β1,3-Ara*f*Gal_2-ABEE	505	860	0.59
larch AGP	29.0	4.99	5.8
arabinan	515	309	1.7
gum arabic	95.7	86.8	1.1

[a]Specific activity was calculated with the calculated molecular mass of 129,964 Da for Bll3HypBA1-NΔ35CΔ761 and 94,145 Da for Bll3HypBA1-NΔ379CΔ761.

[b]Ratio of the specific activities of Bll3HypBA1-NΔ35CΔ761 to Bll3HypBA1-NΔ379CΔ761.

Table S3. X-ray crystallographic data collection and refinement statistics of Bll3HypBA1.

Data set	NΔ379CΔ761 SeMet	NΔ379CΔ761 + Tris	NΔ379CΔ761 Zn peak	NΔ379CΔ761 Zn low remote	NΔ379CΔ933 Ligand free
Data collection[a]					
Beamline	PF-AR NE3A	PF-AR NE3A	PF-AR NW12A	PF-AR NW12A	PF BL17A
Wavelength (Å)	0.9791	1.0000	1.2800	1.3000	0.9800
Space group	$P2_1$	$P2_12_12_1$	$P2_1$	$P2_1$	$P2_1$
Unit cell (Å, °)	a = 59.612, b = 112.325, c = 102.747, β = 96.306	a = 59.592, b = 111.571, c = 153.010	a = 59.582, b = 111.698, c = 113.771, β = 104.776	a = 59.588, b = 111.708, c = 113.778, β = 104.781	a = 75.231, b = 83.510, c = 102.034, β = 90.659
Resolution (Å)	49.21–2.00	49.71–1.75	49.35–2.80	49.35–2.80	49.20–1.70
R_{merge}	0.141 (0.533)	0.176 (1.014)	0.175 (0.445)	0.174 (0.442)	0.076 (0.939)
R_{pim}	0.032 (0.132)	0.102 (0.592)	0.104 (0.264)	0.103 (0.262)	0.048 (0.595)
Total reflections	1847886 (74746)	695814 (34242)	136143 (17948)	136125 (17942)	469771 (22932)
Unique reflections	90717 (4451)	103620 (5038)	35591 (4702)	35597 (4702)	137533 (6721)
Mean $I/\sigma(I)$	20.1 (6.0)	8.9 (2.1)	5.0 (2.3)	5.0 (2.3)	7.3 (1.0)
$CC_{1/2}$	0.998 (0.963)	0.993 (0.696)	0.976 (0.872)	0.976 (0.871)	0.998 (0.676)
Completeness (%)	100.0 (100.0)	100.0 (100.0)	100.0 (100.0)	100.0 (100.0)	99.1 (98.3)
Multiplicity	20.4 (16.8)	6.7 (6.8)	3.8 (3.8)	3.8 (3.8)	3.4 (3.4)
Anomalous	99.7 (99.2)	–	94.4 (91.9)	94.3 (91.8)	–

completeness (%)					
Anomalous multiplicity	10.2 (8.4)	–	1.9 (2.0)	1.9 (2.0)	–
Wilson B (Å^2)	11.52	5.03	13.22	14.03	18.21
Mol/ASU[b]	1	1	1	1	2
Refinement					
Resolution (Å)		49.76–1.75			43.57–1.70
No. of reflections		103529			137521
R_{work}/R_{free}		0.1429/0.1673			0.2202/0.2594
Number of atoms		6216			11092
Residues		M379–V1051			M379–E1050 (A/B)
MolProbity score		1.09			2.16
Clashscore		1.65			1.21
RMSD from ideal values					
Bond lengths (Å)		0.0118			0.0079
Bond angles (°)		1.64			1.43
Ramachandran plot (%)					
Favored		97.02			96.64
Allowed		2.98			3.21
Outlier		0.00			0.15
PDB code	–	8K7X	–	–	8K7Y

[a] Values in parentheses are for the highest resolution shell.

[b] Number of molecules per asymmetric unit.

Table S4. Specific activities of Bll3HypBA1 mutants.

Mutants	Specific activity (Units/mg)	Relative activity[a] (%)
Wild type	3.33	100
E694Q	3.63×10^{-4}	0.01
E694A	1.43×10^{-4}	< 0.01
E723Q	5.49×10^{-3}	0.17
E723A	6.10×10^{-5}	< 0.01
C725S	3.35×10^{-1}	10
C804S	2.12×10^{-4}	< 0.01
C805S	1.71×10^{-4}	< 0.01
E694Q/C804S	ND	ND

[a]Relative activity was expressed as the percentage of the activity against the wild type Bll3HypBA1-NΔ379CΔ933 enzyme.
